# UAV aided virtual cooperative spectrum sensing for cognitive radio networks

**DOI:** 10.1371/journal.pone.0291077

**Published:** 2023-09-05

**Authors:** Noor Gul, Su Min Kim, Jehad Ali, Junsu Kim

**Affiliations:** 1 Department of Electronics, University of Peshawar, Peshawar, Khyber Pakhtunkhwa, Pakistan; 2 Department of Electronics Engineering, Tech University of Korea, Siheung, Gyeonggi, Republic of Korea; 3 Department of AI Convergence Network, AJOU University, Suwon, Republic of Korea; TU Wien: Technische Universitat Wien, AUSTRIA

## Abstract

Cooperative spectrum sensing (CSS) involves multiple secondary users (SUs) reporting primary user (PU) channel sensing states to the fusion center (FC). However, the high overheads associated with multi-user CSS impose power limitations that limit its usefulness in unmanned aerial vehicle (UAV) networks. To address this challenge, we propose a virtual CSS, where a single UAV conducts CSS while following a circular flight trajectory in the air. The novelty of our approach is presenting a working frame structure for the UAV flight, including sensing and data transmission periods with further division of the sensing time into mini-sensing slots. In the virtual CSS, UAV performs local sensing decisions in each mini-slot and accumulates them for a final decision. The proposed virtual CSS scheme exploits sequential decision fusion (SDF), which sequentially adds individual mini-slot decisions. Additionally, we leverage machine learning (ML), employing AdaBoost ensembling classifier (ENC), to inspect flight conditions and reconfigure mini-slot periods dynamically for both traditional decision fusion (TDF) and our proposed SDF schemes. Furthermore, we identify an optimal decision threshold (ODT) for the proposed SDF, enabling the comparison of sequential results with an adjustable threshold through majority voting. This novel approach results in energy efficiency and improved throughput for virtual CSS using SDF, surpassing the performance of TDF, which relies on collecting entire mini-slot reports for its final decision. Simulation results demonstrate the effectiveness of the proposed SDF following the ENCODT (SDF-ENCODT) scheme compared to existing techniques from the literature. We explore varying levels of UAV flight velocities, moving radius, detection probability demand, and channel signal-to-noise ratio (SNR), reinforcing the significance of our contribution. Our research highlights the motivation to address spectrum scarcity in UAV communication by proposing an innovative virtual CSS scheme based on SDF. The proposed approach enhances spectrum utilization, overcomes power limitations, and substantially improves CSS for UAV networks.

## 1 Introduction

Unmanned aerial vehicles (UAVs) have recently attracted the interest of various commercial, public, and industrial applications in a wide range because of their versatility. Integrating UAVs with other wireless technologies and mobile networks can improve spectral efficiency and offer innovative solutions to communication challenges, particularly in beyond fifth-generation (B5G) and sixth-generation (6G) future networks [1, 2]. Furthermore, the high altitude of UAVs leads to line of sight (LOS) channels with the ground nodes, in contrast to the terrestrial channels that suffer from multipath fading, shadowing, and severe path losses [3]. As a result, various applications like military, telecommunication, surveillance, medical supplies delivery, and rescue operations weigh the UAVs as perfect enablers for seamless communications [4]. The UAVs are also expected to be the center of 6G wireless networks, which have gained significant attention from the research community and industry to support emerging mobile services [5].

Nevertheless, UAVs traditionally serve in industrial scientific and medical (ISM) bands, IEEE-S bands, and IEEE-L bands which are permanently exploited by other wireless technologies. It is foreseen that the growing devices force overcrowding of the available spectrum and cause spectrum scarcity issues for the UAVs in the near future [6, 7]. Therefore, spectrum scarcity is an inevitable challenge for UAV-based communication networks. Furthermore, another obstacle for UAV communication is the energy constraints since the limited battery power is the source for flight, hover, and communications [8]. Therefore, UAVs’ integration with cognitive radio (CR) technology can benefit from dynamic spectrum access (DSA) to provide solutions to the problems. The assimilation of the UAVs with CR enables UAVs to exploit and operate in licensed and unlicensed spectrum bands. In this context, among the other goals, spectrum sensing is a crucial function for the CR system, as different spectrum management results rely on sensing performance. A CR-featured device can follow an individual or cooperative spectrum sensing (CSS) mode to detect spectrum availability. In individual sensing, a single secondary user (SU) independently senses the primary user (PU) channel and decides the channel availability. At the same time, in the CSS, all SUs report their local findings for a final decision to the fusion center (FC) [9].

### 1.1 Related work

Numerous studies have been conducted to address the challenges of spectrum sensing in UAV-based cognitive radio networks (CRN). For instance, [1] supplies a spectrum congestion solution using relaying UAVs between the terrestrial PU and SU in the underlay network. A long-term secondary throughput is addressed through fusing adjusted deep deterministic policy gradient (ADDPG) and convex optimization in [10]. The investigation into the influence of sensing energy and data availability on the secondary throughput of an energy-harvesting CRN is outlined in [11]. This study emphasizes using energy harvested from primary signals to power secondary transmitters. A survey in [12] delves into CSS within mobile energy-harvesting CRNs. The article aims to devise an optimal CSS strategy, particularly regarding the final decision threshold *k* to maximize the anticipated achievable network throughput. This scheme is carried out while considering collision and energy causality constraints. In the context of swarm UAVs, [13] suggests a pioneering collaborative search and localization approach. This method relies on a low-complexity Q-learning algorithm and a fast Fourier transformation (FFT)-based location prediction algorithm. Addressing the constraints of software-defined radio (SDR) deployments, [14] contributes by introducing a resource allocation strategy for CRs, specifically those implemented using SDR technology that enhances spectrum utilization. The work in [15] showcases the application of machine learning models to predict spectrum usage within a network. The model leverages a dataset to distinguish between voice and data communication, aiding spectrum prediction. A novel approach to boosting spectrum utilization in cognitive UAV networks is mentioned in [16]. This approach integrates a continuous hidden Markov model with a unique signal-to-noise ratio (SNR) estimation method within a CSS scheme. Likewise, a joint optimization solution is provided in [17] for the overlay operating mode. Here, the CR-enabled UAV performs sensing and tunes to the right frequency by finding the 3-dimensional (3D) location and optimal resource allocation to support the secondary and primary transmission needs. In [18], UAVs assist terrestrial network communication by supervising spectrum sensing operations. The UAV acts as an energy-harvesting sensing node by moving around the PU and delivering information to the destination using the vacant spectrum. The study in [8] has enabled UAVs to adjust the single slot sensing radian to achieve specific detection probabilities. In addition, the assumed energy-harvested UAV-CRN has achieved high spectral efficiency (SE) and energy efficiency (EE) under limited UAV power constraints. Compared with single SU reinforcement learning design studies, a cluster of UAVs in [19] offers collaborative solutions through multi-agent-reinforcement learning (MARL). This includes cognitive UAVs as randomly distributed SUs in the air and the PUs transmitters or opposing jammers in the network. The work in [20] proposes a 3D joint spatial-temporal spectrum sensing in heterogeneous space, which leverages the UAV spectrum sensors’ location flexibility. The discussed literature considers multi-user CSS for the UAV-CR system to improve the throughput and detection performance. However, these studies must consider that sensing PU activity through the multi-user CSS requires extensive communication resources.

Although CSS with multiple sensing nodes provides high sensing performance, reporting delay, control channel bandwidth, and high energy cost limit its persuasiveness for the UAV-CR network. In this regard, multi-slot CSS is proposed in [21–24], which divides the sensing period into many small durations to make local decisions. The local decisions in these sensing slots are combined with the voting rule to make a final decision. However, too many local multi-slot choices also result in high energy consumption, which limits the CSS effectiveness for the UAV-CR environment as the UAV has power limitations. Moreover, as in the well-known virtual CSS, many sensing slots with fixed periods may limit the network performance. Hence fewer sensing decisions with reconfigurable mini-slot duration will increase the data transmission rate, network throughput and energy efficiency. Table 1 summarizes the related works.

**Table 1:** Literature summary of related work.

Research topic on UAV-based sensing	Modeling Approach	References
Spectrum congestion solution with relaying UAV.	Power control procedure compared to numerical methods.	[1]
Enhance energy and spectrum efficiency.	Intra-frame structure-based cognitive UAV-CSS.	[4]
Optimize EE and user outages.	Multi-Agent Reinforcement Learning.	[5]
EE and SE for UAV-CRN.	Delay aware-approximate optimization strategy.	[8]
Optimize the long-term secondary throughput.	ADDPG and convex optimization.	[10]
Impacts of sensing energy and data availability on the secondary throughput.	General data arrival and heavy data arrival processes.	[11]
CSS for mobile energy harvesting CRN.	General *k*-out-of-*M* fusion rule.	[12]
Interference source localization.	Low complexity Q-learning and FFT.	[13]
Alleviation of limitations in SDR deployment.	Markov random field framework.	[14]
Voice or data communication spectrum prediction.	Machine learning algorithm.	[15]
Enhanced spectrum utilization in cognitive UAVs.	Continuous hidden Markov model.	[16]
3D positioning and power allocation.	Joint optimization solution with adapted particle swarm optimization.	[17]
Sensing decision and throughput improvement.	UAV-assisted energy harvested CR relay network.	[18]
Channel access in the clustered cognitive UAV network.	Multi-agent reinforcement learning.	[19]
Spectrum sensing optimization.	3D spatial-temporal spectrum sensing framework.	[20]
CSS in the presence of a Byzantine attack.	Sequential voting rule.	[21]
Energy efficient interweaves UAV-based CSS.	Multi-sensing slot voting scheme.	[22]
CSS with efficient SDF for the terrestrial CRN.	Sequential decision fusion for multiple SUs.	[23]
EE multi-radian overlay sensing in the UAV-CRN.	Alternative Iterative Optimization (AIO) algorithm.	[24]

Energy efficiency and throughput achievements are critical and demanding issues in the UAV-based CR networks. This work addresses these crucial concerns. The significant contributions of this paper can be itemized as follows:

- This paper proposes a virtual cooperative environment with a single UAV serving like multiple SUs in the typical CSS environments. Thus, to achieve a virtual cooperative environment, the frame structure of the flying UAV is divided into sensing (sensing radian) and data transmission time (transmission radian). The sensing time is further divided into mini-slot radians to get high throughput and reduce the number of local decisions which suit the UAV’s power limitations. The UAV performs local sensing in these mini-slots and combines all decisions to form a final decision using the proposed sequential decision fusion (SDF) scheme.
- The proposed SDF scheme sequentially adds binary decisions of the mini-slots and compares them with the adaptive threshold through majority voting in the *k* − *in* − *N* rule. The UAV first reconfigures its mini-slot sensing time using a machine learning (ML) adaptive boosting (AdaBoost) boosted tree ensemble classifier (ENC) by inspecting UAV flight characteristics and sensing demands. These include flight radius, rotation velocity, received SNRs of the channel, detection, and false alarm probability demands. Compared with holding their fixed slot approach, this re-adjustment of the sensing time using ENC improves the throughput and energy efficiency results of the SDF and traditional decision fusion (TDF) schemes.
- Moreover, an optimal decision threshold (ODT) is determined for the SDF through a simple search technique in the *N* mini-slots, which results in low average decisions for the SDF-based virtual cooperative scheme compared to the TDF scheme. The ODT selection improves the throughput and energy efficiency of the proposed SDF cooperative scheme, as the TDF scheme always requires a complete set of mini-slot decisions for its majority voting *k* − *in* − *N*.

## 2 Materials and methods

### 2.1 UAV flight path and frame structure

**Fig 1:**
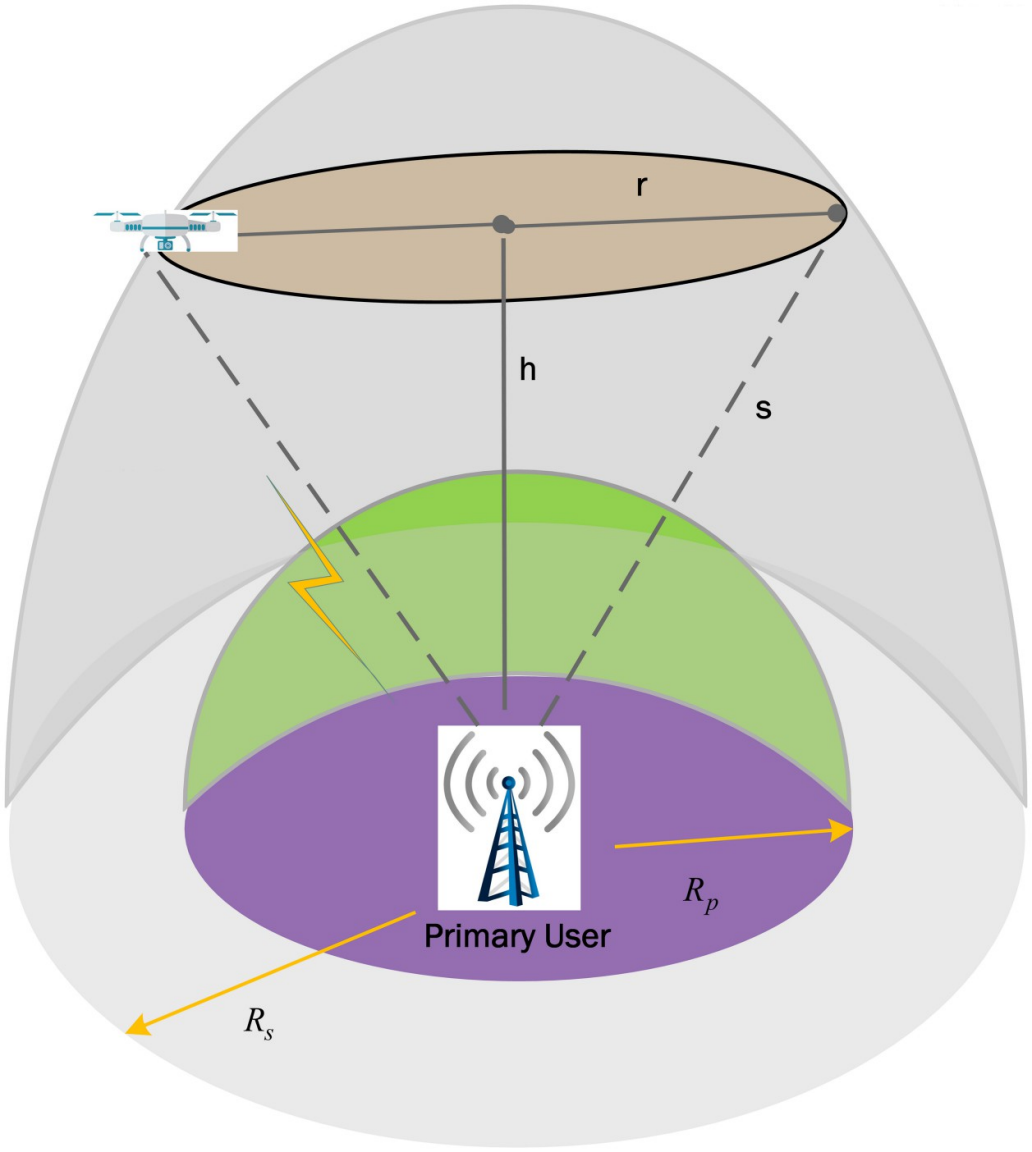
UAV based spectrum sensing model.

This section shows the UAV circular flight path and frame structure to sense the PU spectrum availability in the interweave mode of the UAV-based CRN. In this model, a single UAV plays the role of multiple SUs to achieve an energy-efficient virtual CSS. Fig 1 shows the PU located at the center with UAV following a circular trajectory in the air. The UAV detects the PU channel availability through local spectrum sensing in the sensing radian and starts transmitting during the data transmission if the channel is detected free. As shown in Fig 1, the sensing radius is *R*_*s*_, where the UAV can sense the PU channel. Similarly, to avoid interference with the PU, UAV’s sensing is not allowed inside the protected radius of *R*_*p*_. The UAV detects the PU signal in the 3-dimensional (3D) ring-shaped space and accesses the vacant spectral band.

**Fig 2:**
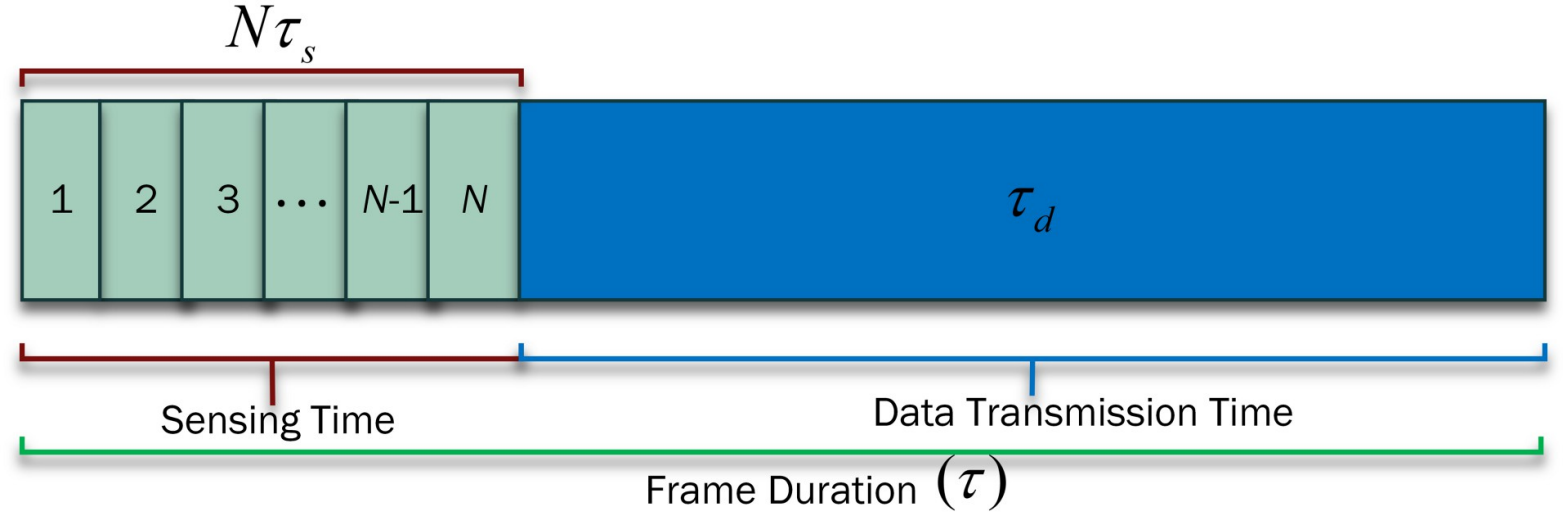
Frame structure with virtual sensing and data transmission time.

**Fig 3:**
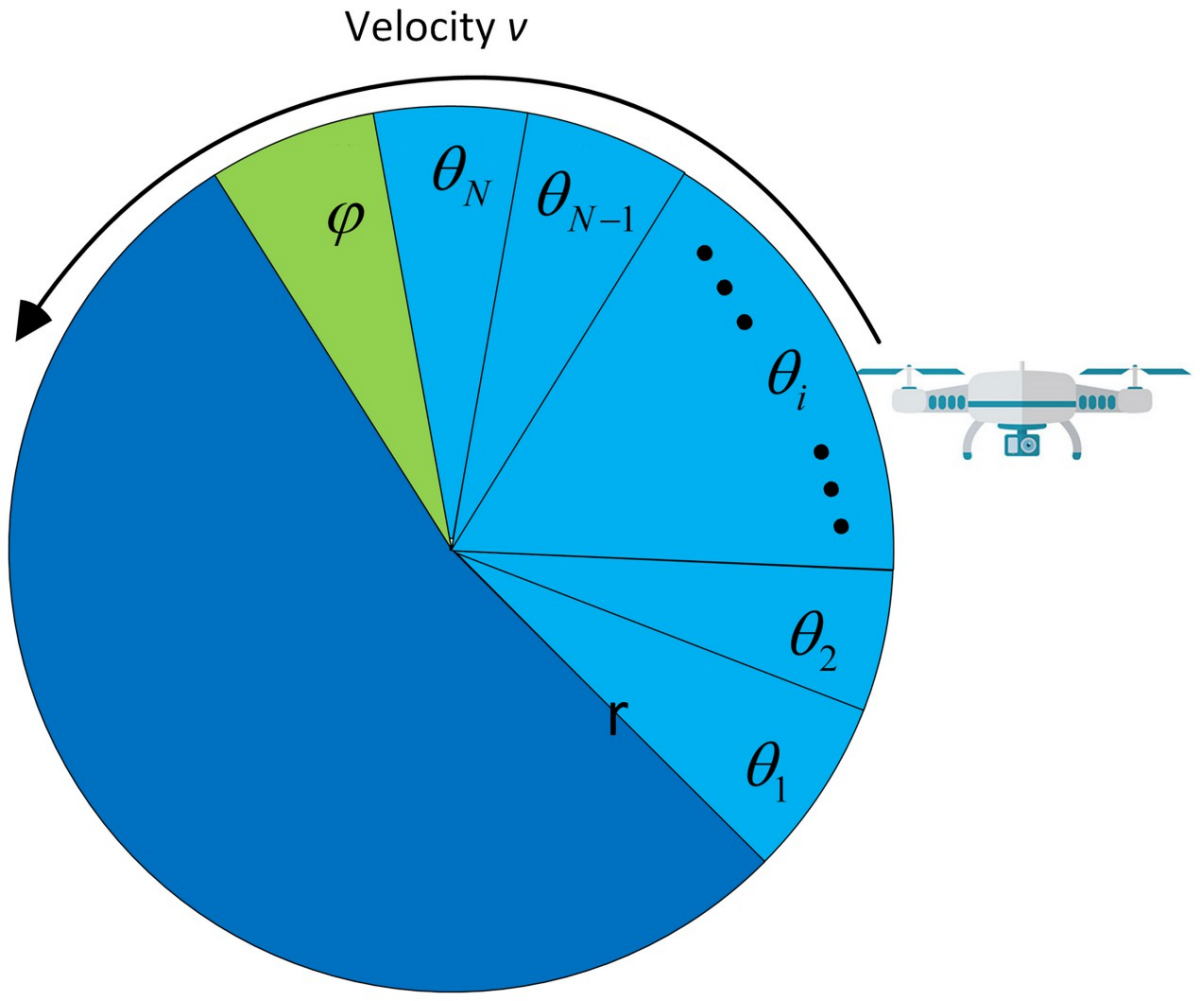
Flight model of the UAV.

As the UAV inside the protected and outside the sensing radius cannot participate in spectrum sensing, they still achieve dynamic spectrum sharing through other relaying UAVs in the network. Fig 2 shows the division of the UAV’s periodic sensing frame structure into sensing time *Nτ*_*s*_ and data transmission time *τ*_*d*_, as in Table 2. Thus, the increase of sensing time is evident with increasing the number of mini-slots *N* or expanding the mini-slot duration *τ*_*s*_. Similarly, rising *τ*_*s*_ lowers the data transmission time *τ*_*d*_ and reduces the UAV’s chances to transmit more data. Fig 3 shows the UAV’s flight around the PU with *τ*_*s*_ and *τ*_*d*_ representation in radian [25]. The UAV is assumed to have a flight radius of *r* with a constant flight altitude *h*. The UAV to PU sensing distance can be expressed as

$$\begin{array}{c} s=\sqrt{r^{2}+h^{2}}, \end{array}$$ (1)

where *R*_*p*_ ≤ *s* ≤ *R*_*s*_.

**Table 2:** List of mathematical notations.

Term	Description
*τ*_*s*,*i*_	*i*^*th*^ mini-slot sensing time
*τ*_*d*_	data transmission time
*τ*	total duration of a frame
*s*_*i*_(*m*)	PU signal in the *i*^*th*^ mini-slot
*E*_*i*_(*y*)	Received signal energy
*Q*(.)	Complementary distribution function
*r*_*i*_	Local decision in the *i*^*th*^ mini-slot
$C_{d}^{tdf}$ , $C_{f}^{tdf}$	TDF cooperative detection and false alarm probabilities
$C_{d}^{sdf}$ , $C_{f}^{sdf}$	SDF cooperative detection and false alarm probabilities
*F*(*q*)	TDF global decision
*ψ*(*N*, *k*, *P*_1_)	Average decisions of the *k* − *in* − *N* with probability *P*_1_
*g*(*q*)	Channel gain of the *q*_*th*_ frame
*T*_0_, *T*_1_	Throughput in the *H*_0_ and *H*_1_ hypothesis
*θ*_*i*_	*i*^*th*^ mini-slot sensing radian
*x*_*i*_	*i*^*th*^ feature vector
*e*_*r*_(*x*_*i*_)	Compound prediction of the *r* classifiers
*h*_*p*_(*x*_*i*_), *α*_*p*_	*p*^*th*^ classifier prediction and weight
*P*(*H*_0_), *P*(*H*_1_)	Probabilities of *H*_0_ and *H*_1_
*Q*^−1^(.)	Inverse complementary Gaussian distribution
*φ*(.)	Negative binomial distribution function

In the UAV-based CR networks, the UAV senses the PU spectrum and utilizes the detected opportunity. In contrast to the ground sensing nodes, the channel from the UAV-based CR to the PU is a LOS. Hence, the UAV sensing system reduces the severe fading effects of terrestrial communication. Therefore, a single UAV can achieve better sensing results than the ground-based CSS with multiple SUs.

### 2.2 Multi-slot virtual cooperative sensing scheme

While a single UAV can establish a LOS connection with the ground PU, there are several limitations with a single UAV for spectrum sensing. These limitations include the UAV’s limited sensing capability due to physical and technical characteristics. Furthermore, interference from other sources, such as other UAVs or ground-based devices, and multipath fading can also cause fluctuations in the received signal strength at the UAV, affecting the accuracy of spectrum sensing [25, 26].

As the energy consumption is high in the conventional multiple nodes CSS, the diversity gain of the single UAV is obtained in the virtual CSS by combining the local measurements from different viewpoints, resulting in a more comprehensive and accurate picture of the spectrum occupancy. Virtual CSS is an energy-efficient cooperative scheme that acquires high sensing performance compared with multi-user cooperation. In the assumed virtual CSS, the flight path consists of the sensing and data transmission radians. The UAV has either to consume more fixed sensing time to make the final decision or to divide the sensing time into multiple sensing slots to make the final decision. A scheme that employs a single sensing and data transmission time slot, with a sensing duration similar to the combined period of *N* multi-slots, provides comparable sensing performance. Still, it has a lower throughput and high energy consumption. Hence, the multi-slot scheme stops sensing for the subsequent sensing slots and switches to another channel for sensing when the PU is detected.

The UAV flight path is shown as a 2-dimensional (2D) plane in Fig 3 with a radius of *r*. For better illustration, the flight path is represented in radians. The virtual cooperative model’s periodic frame structure divides the flight track into *N* sensing radian duration of *θ*_*i*_ and *φ* data transmission radian. Hence, the sensing period of the *i*^*th*^ mini-slot is represented in time for a uniform flight velocity *v* and radius *r* as

$$\begin{array}{c} \tau_{s}=\frac{\theta_{i}r}{v}. \end{array}$$ (2)

For fixed *v* and *r* values, a reconfiguration and careful selection of *θ*_*i*_ leads to the effective values of the sensing time *τ*_*s*_. Similarly, the data transmission duration *τ*_*d*_ at the available channel is given by

$$\begin{array}{c} \tau_{d}=\frac{\varphi r}{v}. \end{array}$$ (3)

Next, the UAV conducts local spectrum sensing in the *N* mini-slots and integrates the decisions for better sensing results. The PU state is assumed to remain stable within *N* mini-slots of the UAV frame structure. Thus, all the virtual mini-slots observe similar PU conditions in the sensing period. Therefore, it is worth mentioning that the PU is believed not to change its state from *H*_0_ to *H*_1_ or *H*_1_ to *H*_0_ in the mini-slots local decisions of a given frame.

#### 2.2.1 Local spectrum sensing

The UAVs locally decide the channel availability in each sensing interval by following the energy detector scheme. The energy detector is considered favored for the given environment because it has no requirements of the PU channel prior knowledge. Furthermore, an energy detector is also less complex and enables effortless implementation. Therefore, the presence and absence states of the PU in the *i*^*th*^ local mini-slot are represented as [27]

$$\begin{array}{c} y_{i}\left(m\right)=\{\begin{array}{cc} u_{i}\left(m\right) & :H_{0}\\ g\left(k\right)s_{i}\left(m\right)+u_{i}\left(m\right) & :H_{1} \end{array}, \end{array}$$ (4)

where *H*_0_ is the absence and *H*_1_ is the present hypothesis of the PU signal. The received PU signal *s*_*i*_(*m*) at the UAV in the *i*^*th*^ mini-slot is distorted by the *q*^*th*^ frame channel gain *g*(*q*). *u*_*i*_(*m*) is the complex Gaussian noise assumed to be independent of *s*_*i*_(*m*) [28].

The UAV receiver is located at a distance of *s* from the PU transmitter, and the signal passes through free space to the UAV. The channel model provides LOS communication and the signal flows along a circular flight, experiencing no obstruction between the PU transmitter and the UAV receiver [29]. Similarly, the channel gains $g\left(q\right)=\frac{\xi }{s}$ is at the unit distance with *ξ* = *c*/(4*πf*_*s*_), where *f*_*s*_ denotes the sampling frequency [30].

The UAV’s received signal energy can be represented as

$$\begin{array}{c} E_{i}\left(y\right)=\sum_{m=1}^{M}{\left|y_{i}\left(m\right)\right|}^{2}, \end{array}$$ (5)

where *M* is the number of samples. According to the central limit theorem, when *M* is sufficiently large, *E*_*i*_(*y*) takes the form as

$$\begin{array}{c} E_{i}\left(y\right)∼\{\begin{array}{cc} N(\mu_{0},\;\;\sigma_{0}^{2}) & :H_{0}\\ N(\mu_{1},\;\;\sigma_{1}^{2}) & :H_{1} \end{array}, \end{array}$$ (6)

where $\mu_{0}=M\sigma_{0}^{2}$ and $\sigma_{0}^{2}=2M\sigma_{0}^{4}$ denote the mean and variance under the *H*_0_ hypothesis while $\mu_{1}=M\left(\gamma +1\right)\sigma_{0}^{2}$, and $\sigma_{1}^{2}=2M{\left(\gamma +1\right)}^{2}\sigma_{0}^{4}$ represent the mean and variance with *H*_1_. $\gamma ={\left|g(k)\right|}^{2}\sigma_{1}^{2}/\sigma_{0}^{2}\;$ is the PU average SNR at the mini-slot of interest.

The mini-slot local sensing energy is compared with the mini-slot decision threshold λ to determine local detection and false probabilities as [31].

$$\begin{array}{cc} P_{f} & =P\left(E_{i}\left(y\right)>\lambda |H_{0}\right)\\ & =Q((\frac{\lambda }{\sigma_{0}^{2}}-1)\sqrt{\frac{r\theta_{i}f_{s}}{v}}) \end{array},$$ (7)

$$\begin{array}{cc} P_{d} & =P\left(E_{i}\left(y\right)>\lambda |H_{1}\right)\\ & =Q((\frac{\lambda }{\sigma_{0}^{2}\left(\gamma +1\right)}-1)\sqrt{\frac{r\theta_{i}f_{s}}{v}}) \end{array},$$ (8)

where *Q*(.) denotes the standard Gaussian complementary distribution function.

### 2.3 Virtual cooperative sensing with TDF rule

In the conventional CSS, the FC collects multiple SUs’ decisions to issue a global decision on the spectrum availability using either the hard or soft fusion rule. In this regard, the *k* − *in* − *N* fusion is a hard straightforward rule, such that if *k* out of *N* decisions from the multiple users declare the PU channel busy, the FC must broadcast the channel busy [32]. On the other hand, TDF is a multi-slot virtual CSS that uses the *k* − *in* − *N* rule for the final decision. The TDF scheme considers similar functionality of the PU channel availability within all mini-slots of the sensing frame for making global decisions. Thus TDF performs local spectrum sensing in these mini-slots and outputs a final binary decision based on the hard *k* − *in* − *N* rule [33]. The global decision *F*(*q*) of the TDF rule following the *k* − *in* − *N* rule is described as

$$\begin{array}{c} F\left(q\right)=\{\begin{array}{cc} \sum_{i=1}^{N}r_{i}<k & :H_{0}\\ \sum_{i=1}^{N}r_{i}\geq k & :H_{1} \end{array}, \end{array}$$ (9)

where *r*_*i*_ is the local decision in the *i*^*th*^ mini-slot. The CSS using the mini-sensing slots exploits the diversity of the virtual sensing and reduces the individual slot sensitivity to improve the detection performance. Therefore, the requirement for a strong FC can be reduced and the sensing cost also can be concentrated in a UAV-CR network [33].

The *k* − *in* − *N* rule is a suitable combination scheme for TDF because of its low complexity with little preliminary information on the PU signal. A fixed number of decisions is always required for the TDF rule regardless of the kind of decision threshold adopted in each frame to make the global decision. Therefore, multi-slot virtual CSS with TDF has improved detection performance at the cost of high control channel overhead, reporting delay, and more energy consumption which restricts the cooperative gain. Because the UAVs have limited battery power, energy efficiency must be considered for the UAV’s virtual cooperative sensing. The final detection $C_{d}^{tdf}$ and false alarm $C_{f}^{tdf}$ probabilities of the TDF based on the *k* − *in* − *N* rule can be presented as

$$\begin{array}{c} C_{f}^{tdf}=\sum_{i=k}^{N}(\begin{array}{c} N\\ i \end{array})P_{f}^{i}{\left(1-P_{f}\right)}^{N-i}, \end{array}$$ (10)

$$\begin{array}{c} C_{d}^{tdf}=\sum_{i=k}^{N}(\begin{array}{c} N\\ i \end{array})P_{d}^{i}{\left(1-P_{d}\right)}^{N-i}. \end{array}$$ (11)

### 2.4 Virtual cooperative sensing with SDF rule

Energy-efficient SDF virtual CSS is the best alternative to reduce the difficulties in the TDF scheme. This modification is encouraged by adding a sequential idea into the TDF to lower the local decisions and improve energy efficiency. The sequential detection scheme achieves almost identical detection performance with minimum average decisions compared with the fixed number of decision schemes [33].

The SDF sequentially processes the local spectrum sensing decisions in each mini-slot until the global decision establishment for the *k* − *in* − *N* rule. The first mini-slot sensing decision *r*_1_ is matched with the voting threshold *k*. Hypothesis *H*_1_ is selected for *r*_1_ higher than *k* and the local sensing process is suspended. Then, the UAV switches to another channel and continues sensing. Otherwise, the UAV employs the data transmission slot to access the channel. Similarly, sensing at the second mini-slot is performed and the decision is made as *r*_2_. The first and the second mini-slot decisions (*r*_1_ + *r*_2_) is compared with the decision threshold *k* to decide either *H*_1_ or *H*_0_. Hence the sensing process continues until ∑(*r*_1_, *r*_2_ ⋯, *r*_*N*_). If the *N* local outcomes are yet lower than *k*, the *H*_0_ global decision is decided; otherwise, *H*_1_ is selected.

Assuming *P*_1_ is the mini-slot probability to state 1, the average number of decisions that satisfy the *k* − *in* − *N* is defined as

$$\begin{array}{c} \psi (N,k,P_{1})=\sum_{i=1}^{N}i\{\phi (i,\;\;k,\;\;P_{1})\\ +\phi (i,\;\;N-k+1,\;\;1-P_{1})\}, \end{array}$$ (12)

where $\phi \left(w,u,p\right)=(\begin{array}{c} w-1\\ u-1 \end{array})p^{u}{\left(1-p\right)}^{w-u}$ represents the probability of the negative binomial distribution function. In the mini-slots global decision, there are either (*k*) 1’s decisions or (*N* − *k* − 1) 0’s findings. Hence,

$$\begin{array}{c} \sum_{i=1}^{N}\phi \left(i,\;\;k,\;\;P_{1}\right)+\phi \left(i,\;\;N-k+1,\;\;1-P_{1}\right)=1. \end{array}$$ (13)

This equation represents the sum of cooperative detection $C_{d}^{tdf}\left(k\right)$ and miss detection $C_{m}^{tdf}\left(k\right)$ probabilities equal to 1. Therefore, fewer average decisions are required in the virtual cooperative sensing with the SDF rule as *ϕ*(*N*, *k*, *P*_1_) ≤ *N*. According to the Bayes theorem, the average decision is represented as

$$\begin{array}{cc} A\left(k\right) & =\phi \left(N,\;\;k,\;\;P_{f}\right)P\left(H_{0}\right)+\phi \left(N,\;\;k,\;\;P_{d}\right)P\left(H_{1}\right)\\ & =(\begin{array}{c} N-1\\ k-1 \end{array})\;P_{f}^{k}{\left(1-P_{f}\right)}^{N-k}P\left(H_{0}\right)\\ & \;+(\begin{array}{c} N-1\\ k-1 \end{array})\;P_{d}^{k}{\left(1-P_{d}\right)}^{N-k}P\left(H_{1}\right), \end{array}$$ (14)

where *P*(*H*_0_) and *P*(*H*_1_) denote the probabilities of the hypothesis of *H*_0_ and *H*_1_. The CSS’s energy efficiency also improves since the SDF involves minimum local decisions to finalize a global decision. Hence, for the case of fixed frame duration *τ*, sensing decision reduction means lowering *A*(*k*)*τ*_*s*_, which leads to the extension of the data transmission duration *τ*_*d*_. The SDF scheme global false alarm and detection probabilities are

$$\begin{array}{c} \;\;\;\;\;C_{f}^{sdf}\left(k\right)=\sum_{i=k}^{N}(\begin{array}{c} i-1\\ k-1 \end{array})\;P_{f}^{k}{\left(1-P_{f}\right)}^{i-k}, \end{array}$$ (15)

$$\begin{array}{c} \;\;\;\;\;C_{d}^{sdf}\left(k\right)=\sum_{i=k}^{N}(\begin{array}{c} i-1\\ k-1 \end{array})\;P_{d}^{k}{\left(1-P_{d}\right)}^{i-k}. \end{array}$$ (16)

In theory, the SDF scheme achieves similar detection and false alarm results to the TDF, for any *k* lies in the range from *k* = 1 to *k* = *N*. However, the SDF dominates the TDF in achieving high throughput performances with vigorous choices to expand its data transmission time. Throughput enhancement will be briefly discussed in the next section.

### 2.5 UAV-CR network throughput

The UAV throughputs *T*_0_ and *T*_1_ in the absence and presence of the PU are formulated as

$$\begin{array}{c} T_{0}={\text{log}}_{2}\;(1+\frac{g{\left(k\right)}^{2}P_{t}}{\sigma_{0}^{2}}), \end{array}$$ (17)

$$\begin{array}{c} T_{1}={\text{log}}_{2}\;(1+\frac{g{\left(k\right)}^{2}P_{t}}{\left(\gamma +1\right)\sigma_{0}^{2}}), \end{array}$$ (18)

where *γ* is the average SNR from the PU to the UAV and *P*_*t*_ denotes the transmit power of the UAV. The UAV can continue to transmit data in both *H*_0_ and *H*_1_ as case 0 and case 1. In *H*_0_, the cooperative decision shows the PU channel is idle when it is free. Hence, there are no false alarm results when the UAV performs spectrum sensing, and the achievable throughput is *τ*_*d*_*T*_0_/*τ*. The *H*_1_ hypothesis shows the PU’s presence while the cooperative decision is idle. Therefore, the miss detection probability appears, and the UAV access interferes with normal PU operations. In this case, the UAV achievable throughput is defined as *τ*_*d*_*T*_1_/*τ*. The probabilities of case 0 and case 1 are given as $(1-C_{f}^{sdf}\left(k\right))\;P\left(H_{0}\right)$ and $(1-C_{d}^{sdf}\left(k\right))\;P\left(H_{1}\right)$. Hence, the case 0 throughput $T_{f}\left(k\right)=\frac{\tau_{d}}{\tau }T_{0}(1-C_{f}^{sdf}\left(k\right))\;P\left(H_{0}\right),$ and case 1 throughput *T*_*d*_(*k*) are derived as

$$\begin{array}{c} T_{f}\left(k\right)=\frac{\tau_{d}}{\tau }T_{0}(1-C_{f}^{sdf}\left(k\right))\;P\left(H_{0}\right), \end{array}$$ (19)

and

$$\begin{array}{c} T_{d}\left(k\right)=\frac{\tau_{d}}{\tau }T_{1}(1-C_{d}^{sdf}\left(k\right))\;P\left(H_{1}\right). \end{array}$$ (20)

Then, the average throughput is given by

$$\begin{array}{c} T\left(k\right)=T_{f}\left(k\right)+T_{d}\left(k\right). \end{array}$$ (21)

In the above equation, the *T*_*f*_(*k*) term on the right dominates the *T*_*d*_(*k*) as *T*_0_ > *T*_1_. In addition, the UAV-based CR may allow an abnormal data transmission of the UAV in *T*_*d*_(*k*) as the UAV and the PU communication interfere. Since *T*_0_ > *T*_1_, the term on the right side of (21) dominates the average throughput. The approximate value of the average throughput is denoted as *T*(*k*), which can be simplified and approximated as

$$\begin{array}{c} T\left(k\right)=T_{f}\left(k\right). \end{array}$$ (22)

The data transmission of the TDF *τ*_*d*_ = *τ* − *Nτ*_*s*_ is of fixed duration as local sensing decisions *N* and *τ*_*s*_ are specified. However, *τ*_*s*_ can be adjusted to maximize TDF throughput. On the other hand, data transmission time in the SDF *τ*_*d*_ = *τ* − *A*(*k*)*τ*_*s*_ is more vibrant than the TDF for each frame. This shows that the average decisions of the SDF scheme depend on *τ*_*s*_ and optimal threshold selection. Therefore, an optimal choice of the decision threshold *k* and sensing time in the virtual cooperative sensing maximizes the achievable throughput of the UAV-CR network for each frame. The throughput optimization problem is mathematically stated as

$$\begin{array}{c} T^{tdf}\left(\tau_{s}\right)=\frac{\tau -N\tau_{s}}{\tau }T_{0}(1-C_{f}^{tdf})\;P\left(H_{0}\right), \end{array}$$ (23)

$$\begin{array}{c} T^{sdf}\left(k,\tau_{s}\right)=\frac{\tau -A\left(k\right)\tau_{s}}{\tau }T_{0}(1-C_{f}^{sdf}\left(k\right))\;P\left(H_{0}\right), \end{array}$$ (24)

where 1 ≤ *k* ≤ *N* and *T*^*tdf*^, *T*^*sdf*^ represent the throughputs of the TDF and SDF schemes.

First, the UAV adjusts its sensing time $\tau_{s}=\frac{r\;\theta_{i}}{v}$ using an ensembled classifier to reconfigure the mini-slot sensing duration in radians (*θ*_*i*_). A reconfiguration of the mini-slots according to UAV rotation features and sensing requirements helps UAVs to adjust the sensing time for improved throughput. Similarly, the well-known TDF scheme depletes consistent local decisions while making a global decision. On the other hand, ODT for the *k* − *in* − *N* of the SDF is selected as the simple search technique that maximizes throughput for a specified sensing time *τ*_*s*_. The throughput improvement is briefly discussed in the following sections.

#### 2.5.1 Sensing time adjustment through ensemble classifier

**Fig 4:**
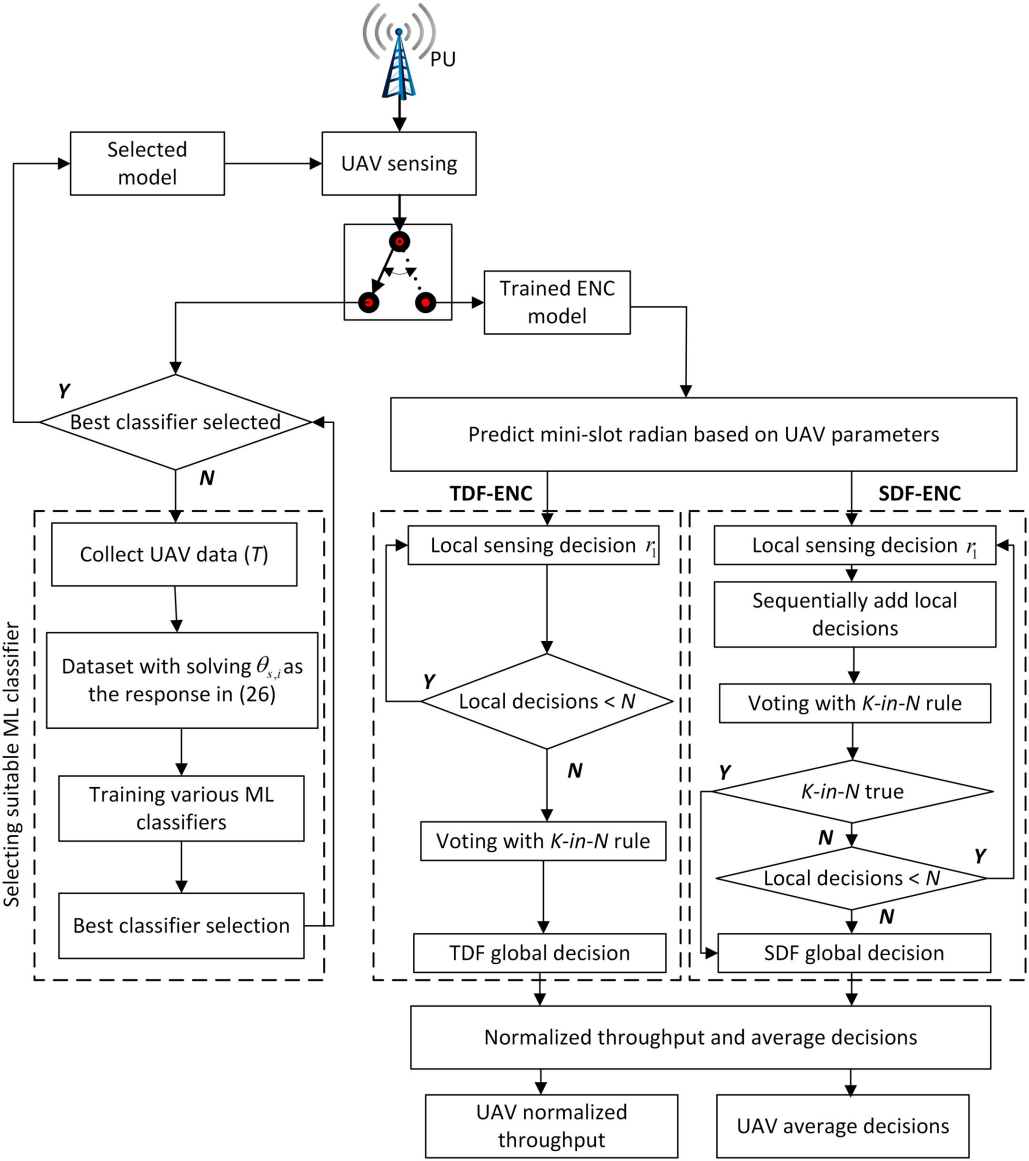
Flowchart of the proposed scheme.

This section reconfigures the mini-slot sensing radians for the UAVs TDF and SDF virtual CSS as in Fig 4. The UAV accomplishes this by considering various flight conditions, such as flight radius, flight velocity, received SNRs, sampling frequency, and mini-slot sensing requirements, such as detection and false alarm probabilities. Eq (8) can be expressed in terms of (7) as follows

$$P_{d}=Q\left(\frac{\sqrt{v}Q^{-1}(P_{f})-\gamma \sqrt{r\theta_{i}f_{s}}}{\left(\gamma +1)(\sqrt{v}\right)}\right).$$ (25)

Solving the above for *θ*_*i*_

$$\begin{array}{c} \theta_{i}={\left\{(\frac{Q^{-1}(P_{f})-(\gamma +1)Q^{-1}(P_{d})}{\gamma \sqrt{rf_{s}}})\sqrt{v}\right\}}^{2}, \end{array}$$ (26)

where *Q*^−1^ (⋅) denotes the inverse complementary Gaussian distribution functions. The optimal sensing angle for a given detection, false alarm, radius, velocity, and SNR requirements in (26) requires the solution of a non-convex function, which is hard to get in real-time. This motivates us to develop a dataset to train the boosted tree ensemble classifier, which uses the AdaBoost ensembling method and to predict the multi-slot sensing radians *θ*_*i*_ further when the model is trained. A configurable value of the sensing angle *θ*_*i*_ adjusts the sensing time as $\tau_{s}=\frac{r\;\theta_{i}}{v}$ according to UAV flight conditions and mini-slot sensing demands.

The result in (26) is first used to create the dataset for training and testing various ML classifiers as

$$\begin{array}{c} T={\left[\begin{array}{cccc} t_{1} & t_{2} & \cdots & t_{n} \end{array}\right]}^{T}, \end{array}$$ (27)

*t*_1_, *t*_2_, and *t*_*n*_ are the *n* feature vectors consisting of the target detection probability *P*_*d*_, false alarm probability *P*_*f*_, SNRs, UAV velocity *v* and flight radius *r* as the input and the sensing radian *θ*_*i*_ as the response to be classified.

This work inspected the training and testing performance of ENC, decision tree (DT), linear regression, random forest classifier (RFC), *k*-nearest neighbor (KNN), neural network (NN), and Gaussian naïve bayes (GNB). The classifier performing better in the training and testing phases is selected and utilized in the virtual CSS to make accurate sensing decisions. The suggested ENC produced better training and testing classification performance and the trained ENC model was chosen to find sensing radians for the TDF and SDF schemes.

The training set *T* is an *n*′ × (*m* + 1) matrix in the space *T* ∈ ℜ^*n*′×(*m*+1)^ as

$$\begin{array}{c} T=[x_{i}|y_{i}],\;i\in \{1,\cdots ,n^{\prime }\}. \end{array}$$ (28)

The input training data to the ensemble classifier in matrix *T* has *X* and *Y* sub-matrices. Here *X* is the *n*′ × *m* feature matrix, and *Y* is the *n*′ × 1 output label matrix consisting of sensing radian information.

ENC is briefly discussed here due to its high classification performance. In the boosted tree ENC with the AdaBoost ensembling technique, each *r*^*th*^ classifier is set with decision weights by knowing the predictions of the *r* − 1 classifiers to express the boosted classifier as

$$\begin{array}{c} e_{r-1}(x_{i})=\sum_{p=1}^{r-1}\alpha_{p}h_{p}(x_{i}),p\in \{1,2,...,r-1\},i\in \{1,...,n^{\prime }\}, \end{array}$$ (29)

where *h*_*p*_(*x*_*i*_) is the *p*^*th*^ classifier prediction and *α*_*p*_ is the weight assigned to the classifier prediction score.

Similarly, the *r*^*th*^ classifier’s prediction is included with *h*_*r*_(*x*_*i*_) as a predicted value and *α*_*r*_ optimum weights to form a better-boosted classifier as

$$\begin{array}{c} e_{r}\left(x_{i}\right)=\sum_{p=1}^{r-1}\alpha_{p}h_{p}\left(x_{i}\right)+\alpha_{r}h_{r}\left(x_{i}\right). \end{array}$$ (30)

Using the value from (29), the above can be written as

$$\begin{array}{c} e_{r}\left(x_{i}\right)=e_{r-1}\left(x_{i}\right)+\alpha_{r}h_{r}\left(x_{i}\right), \end{array}$$ (31)

where *e*_*r*_ (*x*_*i*_) is the compound predicted value obtained by grouping the *r* classifiers. We are interested in a closed-form formula for *α*_*r*_, which assigns *α*_*r*_ a value such that the total error of prediction is minimized

$$\begin{array}{c} \alpha_{r}=\frac{1}{2}\text{ln}\;(\frac{W_{c}}{W_{e}}). \end{array}$$ (32)

Because *W*_*c*_ = *W* − *W*_*e*_, where *W* is the total sum of the weights, therefore

$$\begin{array}{c} \alpha_{r}=\frac{1}{2}\text{ln}\;(\frac{W-W_{e}}{W_{e}}). \end{array}$$ (33)

The expression for the weight *α*_*r*_ in the final form is

$$\begin{array}{c} \alpha_{r}=\frac{1}{2}\text{ln}\;(\frac{1-e_{m}}{e_{m}}), \end{array}$$ (34)

where $e_{m}{=}^{W_{e}}{/}_{W}$ is the weighted error rate of the weak classifier *h*_*r*_.

The UAV using well-known virtual CSS is manually tuned to a suitable velocity and flight radius to get higher throughput and reduce sensing decisions while deciding the PU channel availability. On the other hand, UAVs adjust their sensing slot durations according to any variations in the flight velocity, flight radius, and SNRs of the channel while following machine learning with the AdaBoost ENC scheme. The duration of the sensing slot remains constant until there is a modification in the UAV’s characteristics. Thus, when either parameter of the UAV changes, the ENC will automatically readjust its mini-slot duration, which in turn modifies the sensing time accordingly. The trained ENC estimate *θ*_*i*_ as *y*_*i*_, which adjusts the sensing time $\tau_{s}=\frac{r\;\theta_{i}}{v}$. The UAV then employs the modified time in the mini-slot of TDF and SDF schemes. This ability is essential for the effective and efficient operation of the virtual CSS in dynamic environments when the UAV characteristics are changed.

#### 2.5.2 Decision threshold computation

The ODT computation in this section will further improve the SDF throughput for a given *τ*_*s*_ of ENC estimation. The ODT is determined by taking the SDF throughput derivative in (24) for *k* as

$$\begin{array}{c} T_{k}^{{}^{\prime }}\left(k,\tau_{s}\right)=T^{sdf}\left(k+1,\tau_{s}\right)-T^{sdf}\left(k,\tau_{s}\right), \end{array}$$ (35)

$$\begin{array}{cc} & T_{k}^{{}^{\prime }}\left(k,\tau_{s}\right)=\frac{\tau -A\left(k+1\right)\tau_{s}}{\tau }T_{0}(1-C_{f}^{sdf}\left(k+1\right))P(H_{0})\;\\ & \;\;\;\;\;\;\;\;\;\;\;\;\;\;\;-\frac{\tau -A\left(k\right)\tau_{s}}{\tau }T_{0}(1-C_{f}^{sdf}\left(k\right))P(H_{0}). \end{array}$$ (36)

The above $T_{k}^{{}^{\prime }}\left(k,\tau_{s}\right)>0$ is equivalent to the following

$$\begin{array}{c} T_{k}^{{}^{\prime }}\left(k,\tau_{s}\right)=((\tau -A\left(k+1\right)\tau_{s})(1-C_{f}^{sdf}\left(k+1\right))\;-(\tau -A\left(k\right)\tau_{s})(1-C_{f}^{sdf}\left(k\right)))>0. \end{array}$$ (37)

For a fixed frame duration, assuming $\tau =\hat{N}\tau_{s}$ with $\hat{N}>N$, $T_{k}^{{}^{\prime }}\left(k,\tau_{s}\right)$ can be expressed as

$$\begin{array}{c} T_{k}^{{}^{\prime }}\left(k,\tau_{s}\right)=(\begin{array}{c} (C_{f}^{sdf}\left(k+1\right)-1)A\left(k+1\right)\tau_{s}\\ +(1-C_{f}^{sdf}\left(k\right))A\left(k\right)\tau_{s}+(C_{f}^{sdf}\left(k\right)-C_{f}^{sdf}\left(k+1\right))\hat{N}\tau_{s} \end{array})>0. \end{array}$$ (38)

The above formula states that $T_{k}^{{}^{\prime }}\left(k,\tau_{s}\right)>0$ for a known value of the sensing time *τ*_*s*_ according to assumption $\hat{N}>N$. Similarly, *T*^*sdf*^(*k*, *τ*_*s*_)is also an increasing function of the decision threshold *k*. As the maximum possible value of *k* is *N*, therefore, maximum *T*^*sdf*^(*k*, *τ*_*s*_) is achieved at *k* = *N*. The optimal decision threshold is determined as *k* = *N*. However, the optimal decision threshold *k* in closed-form expression is unavailable and a search over all possible values is required. Thus, it is concluded that since *k* is an integer in the 1 < *k* < *N* range, it is easy to search for the decision threshold *k* [24, 34].

## 3 Simulation results and discussions

Intensive simulation is performed to get the results for simple SDF and TDF, along with ENC and ODT (ENCODT)-based TDF and SDF schemes. Furthermore, we confirm the accuracy and efficiency of our theoretical evaluations regarding the attainable throughput for UAV-based cognitive radio systems. The PU channel state is assumed to remain constant in the multi-slots of a given frame. The simulation network comprises a single PU and UAV with a CR feature. The UAV is supposed to rotate around a fixed location PU at a constant height. The PU transmission power in the network is stable. Table 3 shows the simulation parameters with a hypothesis probability of 0.5, frame duration of 600sec, UAV height of 200m, and 20 mini-slots. The frames counted for the UAV sensing are 10^5^ with a sampling frequency of 600MHz. The mini sensing radian for the SDF and TDF without ML is 1/2*π*. The sensing radian is then predicted through the trained ENC in the TDF-ENC and SDF-ENCODT schemes. We tested various ML classifiers to find their suitability for improving the SDF and TDF performance. The mini sensing radian for the SDF and TDF without ML is 1/2*π*. The sensing radian is then predicted through the trained ENC in the TDF-ENC and SDF-ENCODT schemes. We tested various ML classifiers to find their suitability for improving the SDF and TDF performance. Table 4 summarizes the simulation scenarios in the result section.

**Table 3:** Simulation parameters.

Parameter	Term	Value
Hypothesis probability	*P*(*H*_0_)/*P*(*H*_1_)	0.5
Sensing frame	*q*	10^5^
Frame duration	*τ*	600s
UAV height	*h*	200m
Sampling frequency	*f*_*s*_	600MHz
Number of mini-slots	*N*	20
Sensing radian of TDF and SDF without ML	*θ*	1/2*π*
Energy threshold	λ	1.008
Noise standard variance	*σ*_0_	1
Range of velocity in the dataset	*v*	5m/s ∼ 30m/s
Range of the flight radius in the dataset	*r*	150m ∼ 500m
SNRs in the dataset	*γ*	−20dB ∼ −10dB
Detection probability in the dataset	*P*_*d*_	0.5 ∼ 1.0

**Table 4:** Simulation scenarios.

Section	Scenario	Sub-Scenario
3.1.	Creating the dataset and ML classifier selection.	• Micro average training performance of the classifiers.
		• Micro average testing performance of the classifiers.
3.2.	Normalized throughput.	• Relation with decision threshold.
		• Relation with flight velocity.
		• Relation with detection probability.
		• Relation with SNRs.
3.3.	Average number of decisions.	• Relation with flight velocity.
		• Relation with decision threshold.
		• Relation with detection probability.
		• Relation with SNRs.
3.4.	Cooperative sensing decision.	• Relation with flight radius.
		• Relation with SNRs.
		• Relation with flight velocity.

### 3.1 Creating dataset and ML classifier selection

Our study involves creating a dataset using the results obtained from (26), which was used to train and test various machine learning classifiers. The main objective of this approach is to identify a classifier that demonstrates high performance in both the training and testing phases, with the ultimate goal of predicting mini-slot sensing radians accurately. To construct the dataset, we measure the mini-slot duration in radians for different values of the UAV flight velocity *v*, UAV flight radius *r*, received signal-to-noise ratios (SNRs) *γ*, detection probability *P*_*d*_, and false alarm probability *P*_*f*_. We generate the dataset by varying the velocity values between 5(m/s) and 30(m/s), with steps of 5(m/s) between each value. The UAV flight radius varies in increments of 20m, ranging from 150m to 500m. Similarly, the SNRs are adjusted at intervals of 1dB between -20dB and -10dB. The detection probabilities vary from 0.5 to 1 in steps of 0.04. We keep the sampling frequency constant at 600MHz, and the program computes sensing radians for different values of these variables within the specified ranges. This results in 35, 000 input and output features (*θ*_*i*_). To evaluate the performance of different classifiers and determine the most suitable one for the given problem, we initially randomize the data and then partition the dataset into 80% training and 20% testing. We categorize the data into training and testing sets to select a suitable classifier that yields high classification performance.

**Fig 5:**
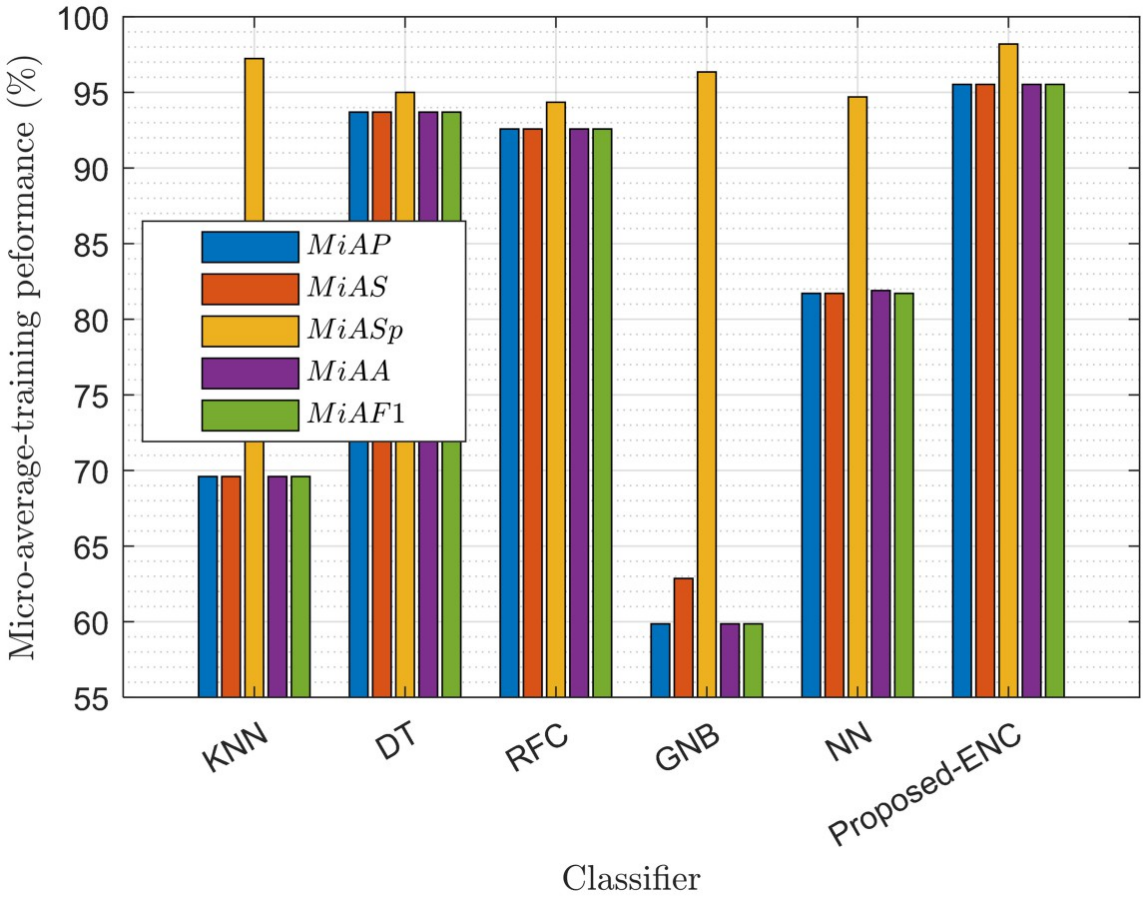
Training micro average performance of the classifier.

**Fig 6:**
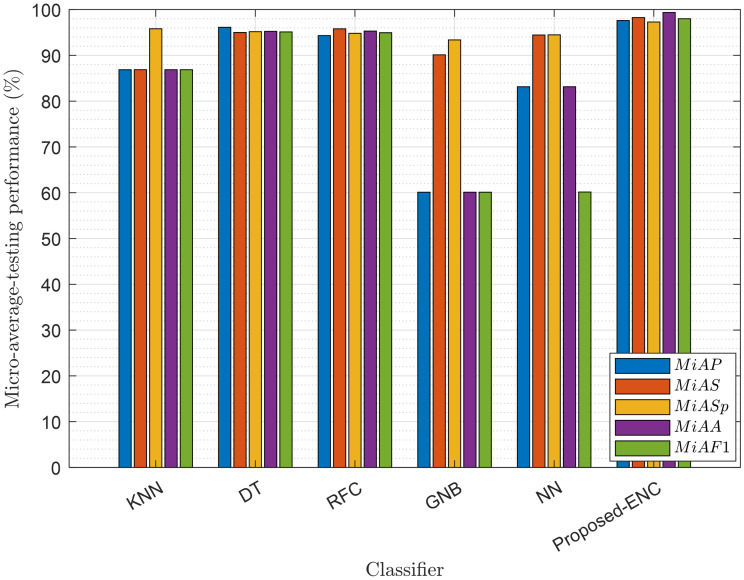
Testing micro average performance of the classifier.

We checked the performance of DT, KNN, RFC, NN, ENC, and GNB classifiers in MATLAB and Python. In the ENC, AdaBoost is employed with decision tree learning. The learner rate is set to 0.1, with maximum splits being 20 and the total number of learners 30. The NN tested in this model has three fully connected layers, with a layer size equal to 10, and uses the rectified linear unit (ReLU) activation function. The maximum number of iterations in the NN is set to 1000. The RFC has a forest of 100 trees, with Gini as the split criteria. The KNN algorithm uses the euclidean distance metric to measure the distance between the test and training points. Finally, the DT used in this model has a maximum split of 100 and uses the Gini diversity splitting criteria. Figs 5 and 6 show the training and testing performance of the classifiers in terms of micro-average-precision (*MiAP*), micro-average-sensitivity (*MiAS*), micro-average-specificity (*MiASp*), micro-average-F1 (*MiAF*1) score, and micro-average-accuracy (*MiAA*) [35–37].

The ENC produces better precision, sensitivity, accuracy, and F1 scores than the other ML classifiers to predict the mini-slot sensing radian in training and testing. The trained ENC model further enables the UAV to adjust sensing radians according to its flight velocity, flight radius, SNRs of the channel, target detection, and false alarm probability demands. The estimated radian measures the throughput and average decisions for the TDF-ENC and SDF-ENCODT schemes in the following sections.

Figs 7–14 in the following section show the normalized throughput performance for the traditional SDF, TDF, SDF-ENCODT, and TDF-ENC schemes against changes in the UAV flight velocity, decision threshold, target detection probability, and SNRs.

**Fig 7:**
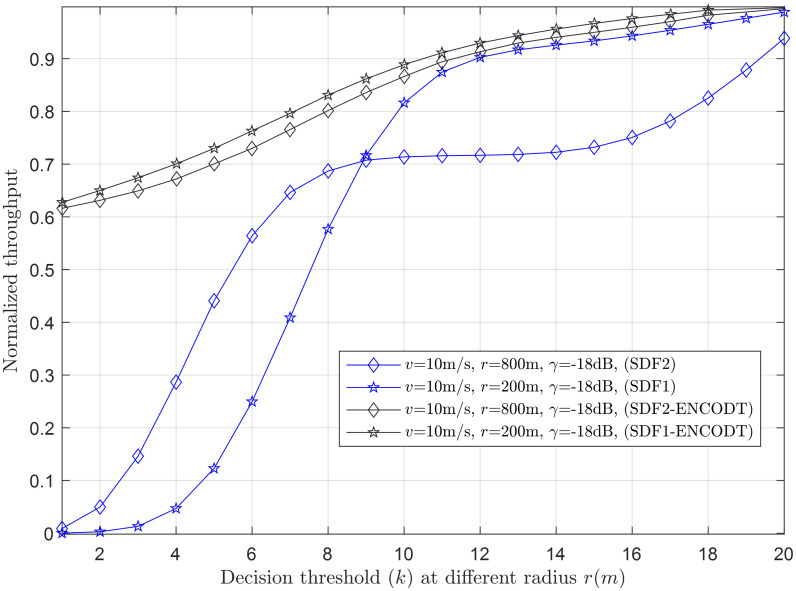
Normalized throughput vs. decision threshold at *r* (200m, 800m), *v* (10m/s), *γ* (-18dB).

**Fig 8:**
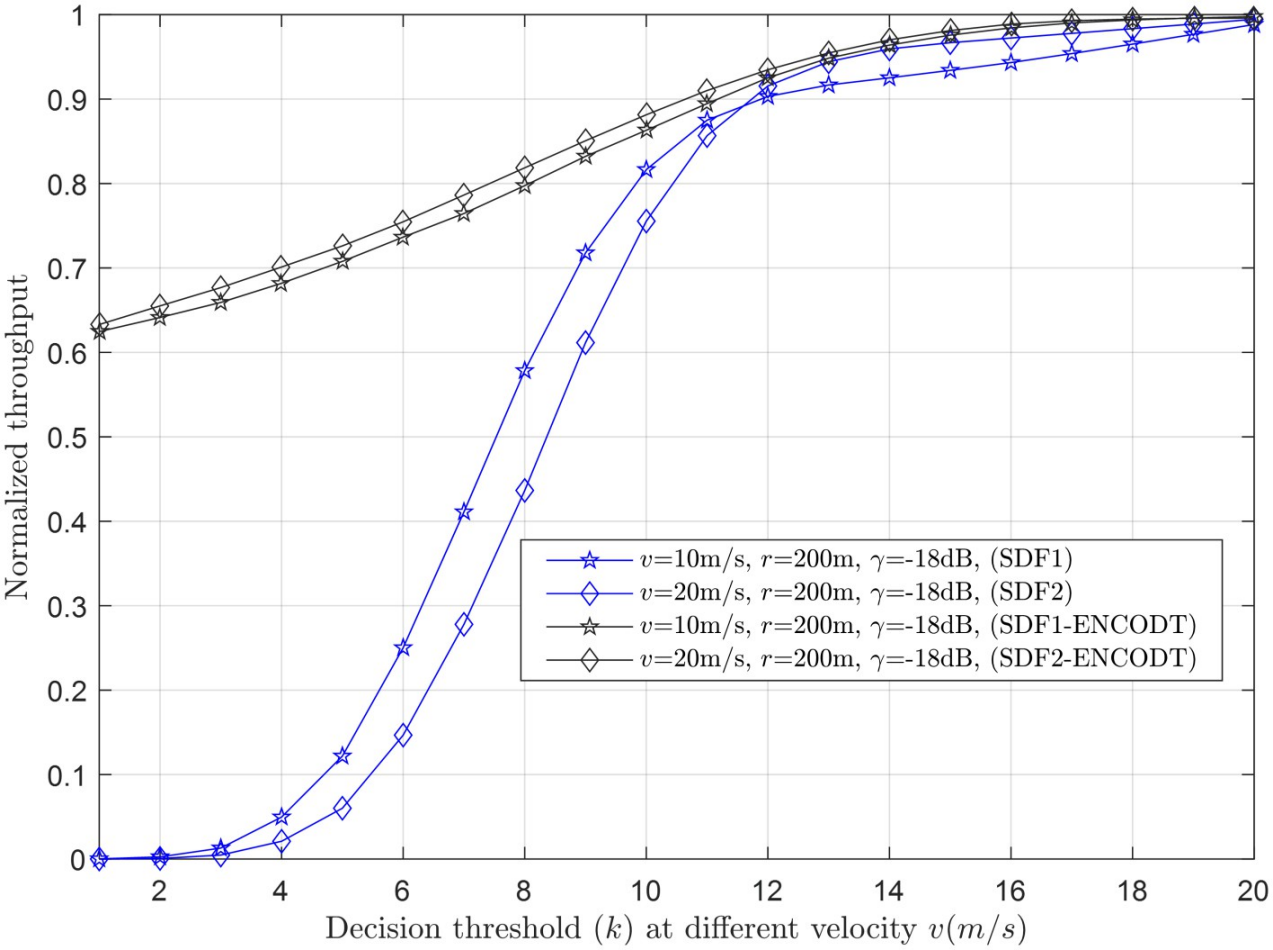
Normalized throughput vs. decision threshold at *v* (10m/s, 20m/s), *r* (200m), *γ* (-18dB).

**Fig 9:**
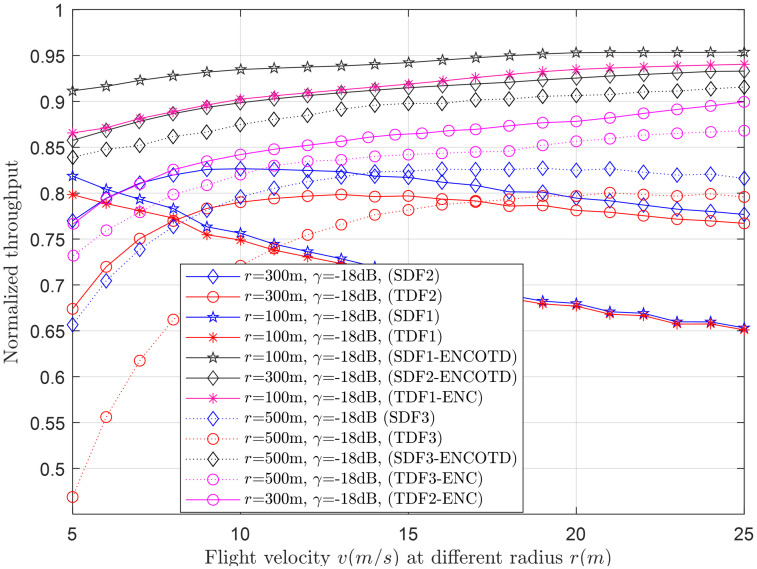
Normalized throughput vs. flight velocity at *r* (100m, 300m).

**Fig 10:**
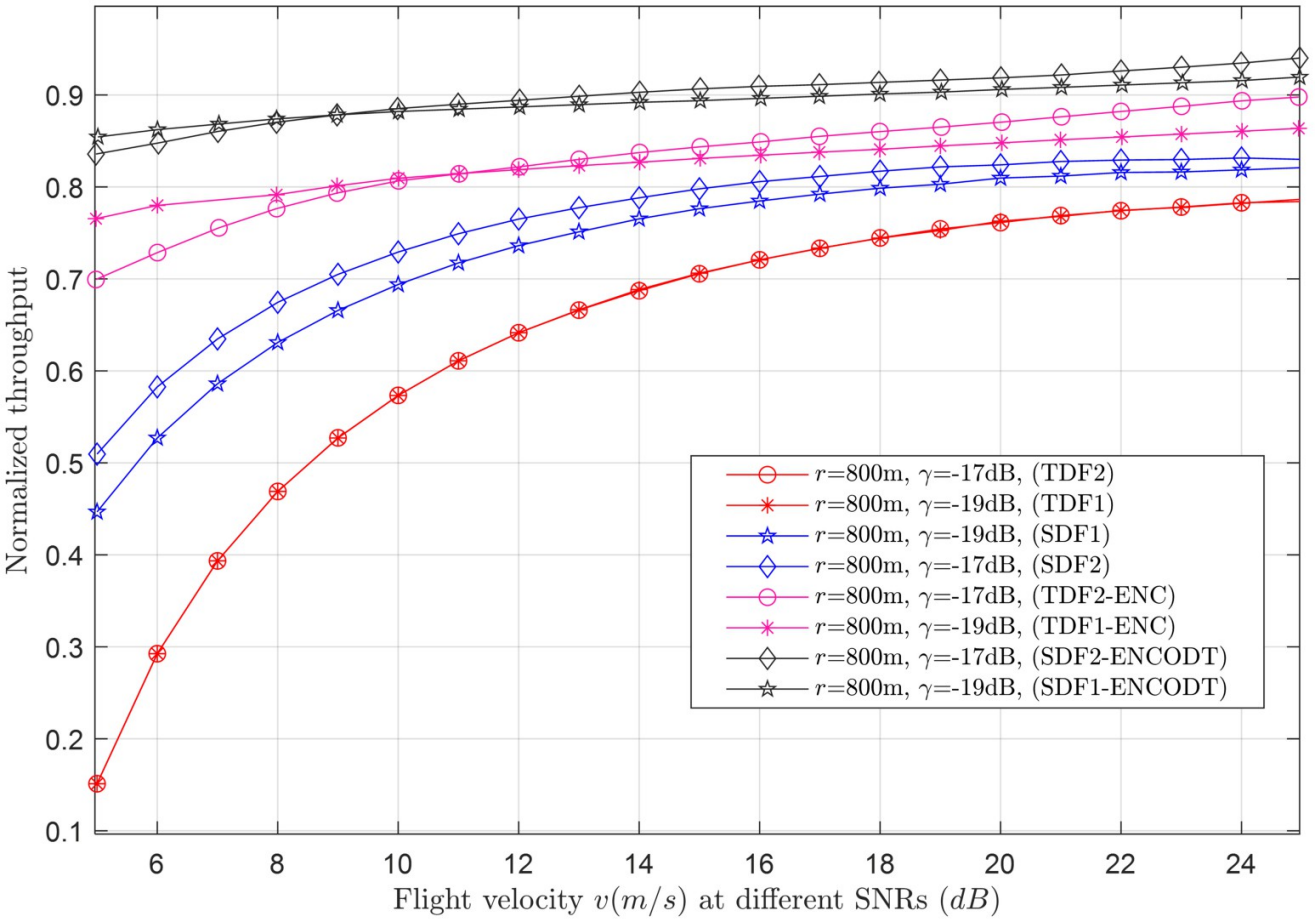
Normalized throughput vs. flight velocity at *γ* (-19dB,-17dB).

**Fig 11:**
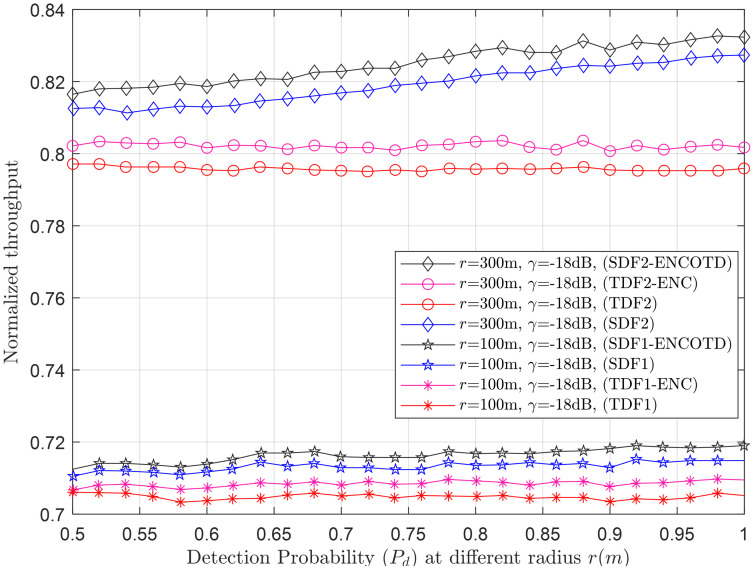
Normalized throughput vs. detection probability at *r* (100m, 300m).

**Fig 12:**
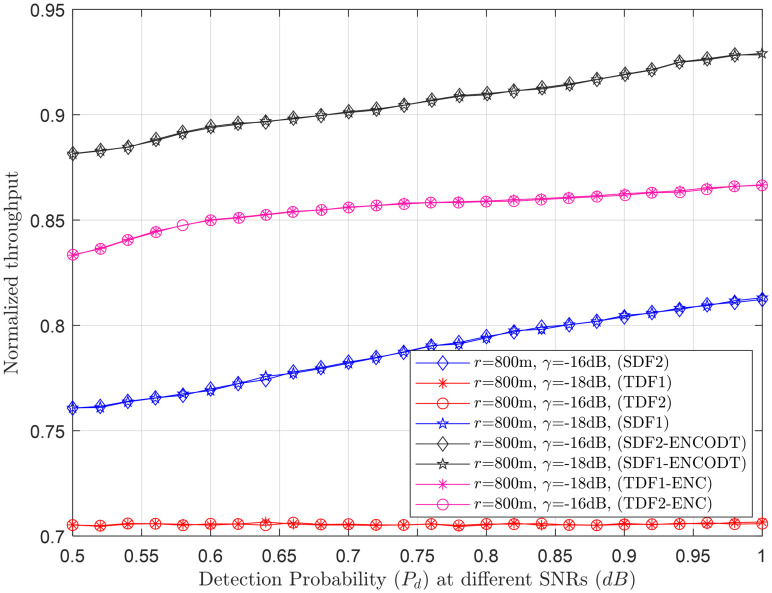
Normalized throughput vs. detection probability at *γ* (-18dB, -16dB).

**Fig 13:**
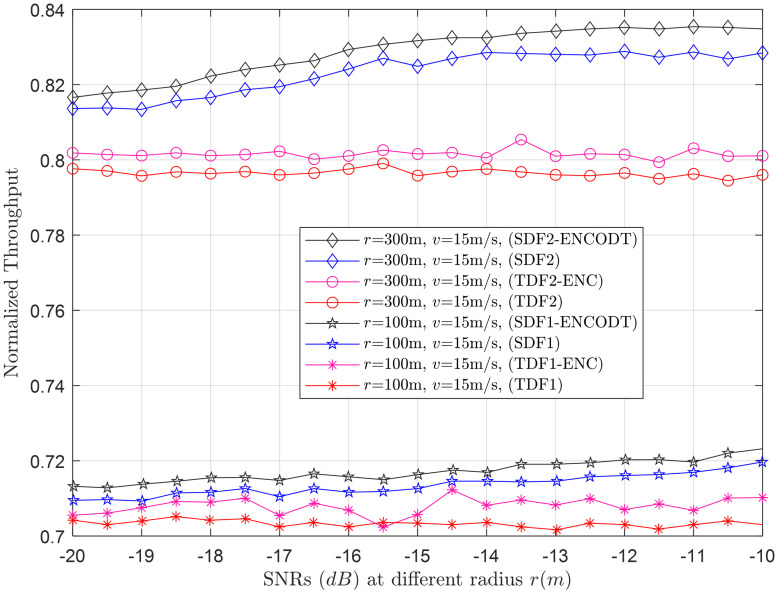
Normalized throughput vs. SNRs (dB) at *r* (100m, 300m).

**Fig 14:**
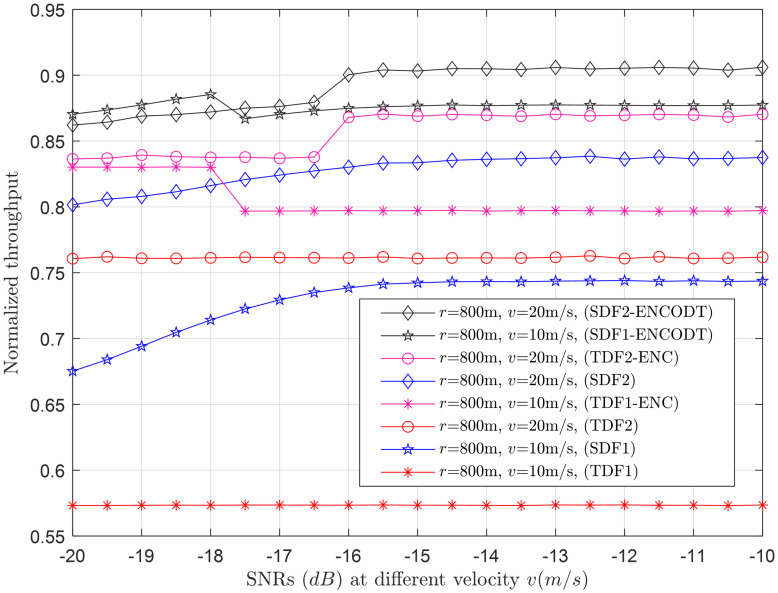
Normalized throughput vs. SNRs (dB) at *v* (10m/s, 20m/s).

### 3.2 Normalized throughput

The decision threshold, rotation velocity, detection probability, and SNRs relation with normalized throughput are briefly discussed in this section. In the assumed virtual CSS, the mini-slot sensing time is $\tau_{s}=\frac{r\;\theta_{i}}{v}$. In the traditional virtual CSS approach with a fixed sensing radian *θ*_*i*_, the sensing radius and UAV velocity need careful adjustments and tuning to achieve an optimal sensing time. Without this adjustment, the tradeoff problem may not exist in scenarios where *r* = 100m and SNR=-18dB or *r* = 800m and SNR varies from -17dB to -19dB. The results in Figs 7–14 show that the sensing radius and flight velocity significantly impact throughput, while the SNR minimally affects the throughput of simple SDF and TDF schemes. However, TDF-ENC and SDF-ENCODT provided higher throughput than TDF and simple SDF schemes, as they adjust the slot sensing radian to compensate for changes in flight radius, flight velocity, and SNRs.

#### 3.2.1 Normalized throughput relation with a decision threshold

Normalized throughput relation with the increase in decision threshold is shown for the simple SDF and SDF-ENCODT in Figs 7 and 8. As the TDF has a fixed number of decision thresholds (*k*), the comparison is shown for the SDF and SDF-ENCODT schemes. Fig 7 shows the throughput against the decision threshold increase at two flight radius levels, *r*_1_ (200m) and *r*_2_ (800m). The figure shows an increase in throughput with increasing decision thresholds. Thus, the maximum throughput according to the ODT is determined to be at *k* = *N*. The figure shows lower throughput of the simple SDF at *r*_2_ than *r*_1_ when the decision threshold crosses the critical point. The SDF-ENCODT has almost identical throughput results at *r*_1_ and *r*_2_, compensating for the changing radius by adjusting the sensing radian. A similar result is shown for the increase in decision threshold at different flight velocities *v*_1_ (10m/s) and *v*_2_ (20m/s) in Fig 8. This shows a higher throughput result of the SDF at *v*_1_ initially, which is then reversed as the decision threshold passes the critical value of the decision threshold. Figs 7 and 8 show higher throughput performance for the proposed SDF-ENCODT schemes than the simple SDF without ENCODT.

For both UAV flight radius (*r*_1_=200m and *r*_2_=800m) and rotation velocities (*v*_1_=10m/s and *v*_2_=20m/s), the SDF-ENCOTD scheme consistently outperforms the SDF scheme in normalized throughput. This indicates that the SDF-ENCOTD scheme is more efficient in utilizing available channel resources, resulting in higher throughput than the basic SDF scheme. Noticeably, the normalized throughput reaches a saturation point as the decision threshold increases (from 1 to 20) for both SDF1 and SDF2 schemes. Beyond this point, further increasing the decision threshold yields slight throughput improvement. Overall, the results in Figs 7 and 8 show the trade-offs between UAV flight radius, rotation velocity, and decision threshold on the performance of the SDF and SDF-ENCOTD schemes. It highlights the importance of optimizing these parameters based on specific use cases and environmental conditions to achieve the best throughput in UAV communication systems.

#### 3.2.2 Normalized throughput relation with flight velocity

This section shows the normalized throughput relation with increasing flight velocity at different levels of the UAV radius and SNRs. Fig 9 shows a consistent decrease in throughput performance of both the TDF and SDF schemes when the flight radius is *r*_1_(100m). Suppose the flight radius is increased to *r*_2_(300m). In that case, it initially has minimum throughput performance when the flight velocity is below a specific limit. It surpasses the throughput performance of *r*_1_ (100m) as the flight velocity reaches a particular limit. Similarly, the throughput results of the TDF and SDF are the lowest of all at *r*_3_ initially for lower speeds which surpasses the throughput performance of *r*_1_ and *r*_2_ as it crosses specific velocity ranges. Another interesting observation is with increasing flight radius. The TDF and SDF scheme throughput shows a more consistent and constant increase/trend in the throughput results with more clear distinction in the outcomes of SDF and TDF schemes. The throughput performance of SDF-ENCODT and TDF-ENC are higher and shows consistent rise, with minimal effects of the increasing flight radius. Fig 10 shows the increase in throughput performance against increasing flight velocity at different levels of the *γ*, such as *γ*_1_ (-19dB) and *γ*_2_ (-17dB). Fig 9 shows higher throughput performance by the SDF-ENCODT scheme at *r*_1_ and *r*_2_. Fig 10 shows higher throughput results of the TDF-ENC and SDF-ENCODT schemes at *γ*_1_ and *γ*_2_.

The throughput values are higher for SDF2 than SDF1 in Fig 9 due to better signal conditions at the larger flight radius. The values are also higher than TDF1 due to the improved signal conditions at a larger flight radius and higher SNR. SDF3 follows a decreasing trend with increasing velocity, similar to SDF1 and SDF2. The throughput values are slightly lower than SDF2, which could be attributed to the larger flight radius. TDF3 throughput shows a decreasing trend with flight velocity, similar to other schemes. The values are lower than TDF1 and TDF2, likely due to the larger flight radius leading to more signal attenuation. In Fig 10, SDF1 throughput decreases with increasing flight velocity, consistent with the observations in Fig 9. Similar to SDF1, SDF2 throughput decreases with increasing speed. The values are higher than those in Fig 9, possibly due to the lower SNR in this scenario. TDF1 throughput generally increases with flight velocity, opposite the trend in Fig 9. This could be due to differences in channel conditions and noise levels. Similar to TDF1, TDF2 throughput increases with velocity, contrary to the observations in Fig 9. This could be due to the different channel conditions and noise levels at this larger flight radius. In summary, the normalized throughput generally decreases with increasing flight velocity for SDF and TDF schemes. However, the trends can vary based on the flight radius and received SNR.

#### 3.2.3 Normalized throughput relation with *P*_*d*_

This section shows the normalized throughput performance of the TDF, SDF, TDF-ENC, and SDF-ENCODT against increasing target detection probabilities at different radii (*r*_1_=100m, *r*_2_=300) and *γ* (*γ*_1_=-18dB, *γ*_2_=-16dB) in Figs 11 and 12. Fig 11 shows lower throughput achievements of the TDF, SDF, TDF-ENC, and SDF-ENCODT against rising detection probability at *r*_1_ (100m). At *r*_2_, a high rise can be seen in the throughput of SDF, TDF, SDF-ENCODT, and TDF-ENC. The schemes achieve higher throughput results at *r*_2_ as compared with *r*_1_. The SDF-ENCODT scheme can also dominate all other schemes at *r*_1_ and *r*_2_ in Fig 11.

Normalized throughput results are next shown for the *γ*_1_ (-18dB) and *γ*_2_ (-16dB) in Fig 12. This indicates that increasing the *γ* from *γ*_1_ to *γ*_2_ does not affect normalized throughput for any given target detection probability increases. Fig 12 shows better throughput performance for the TDF-ENC and SDF-ENCODT schemes than simple TDF and SDF rules for increasing target detection probability.

In Fig 11, each scheme (SDF1, SDF2, TDF1, TDF2, SDF1-ENCOTD, SDF2-ENCOTD, TDF1-ENC, TDF2-ENC) is associated with a specific combination of UAV flight radius and received SNR. We can analyze the performance of these schemes under different conditions. In Fig 11, SDF1 and SDF2 show relatively higher throughput performance than TDF1 and TDF2 schemes, especially at higher detection probability levels (*P*_*d*_> 0.8). The SDF1-ENCOTD and SDF2-ENCOTD schemes show performance improvement over their respective non-ENCOTD counterparts (SDF1 and SDF2) for specific values of *P*_*d*_. The performance of the schemes can vary based on the UAV flight radius and received SNR. Fig 12 compares schemes with a fixed UAV flight radius (*r*=800m) and different received SNRs (*γ*_1_=-18dB and *γ*_2_=-16dB). The performance of SDF1, SDF2, and TDF1 remains relatively consistent between the two SNRs. However, TDF2 shows a slight drop in detection probability. The schemes with “ENCOTD” in their names (SDF1-ENCOTD, SDF2-ENCOTD) again show improvement over their non-ENCOTD counterparts (SDF1 and SDF2) at various *P*_*d*_ levels and SNRs. Based on the analysis in Figs 11 and 12, SDF consistently perform better than the simple TDF schemes at higher detection probability levels (*P*_*d*_> 0.8). Therefore, these schemes may be more suitable for scenarios where high detection probability is crucial. If the UAV flight radius is fixed at 800m, SDF1 and SDF2 remain strong contenders, and TDF may be avoided due to its slightly lower performance.

#### 3.2.4 Normalized throughput relation with SNRs

This section illustrates the normalized throughput performance for the increase in *γ* at the sensing mini slots. Fig 13 shows the throughput performance at *r*_1_ (100m), *r*_2_ (300m) and fixed velocity *v* (15m/s). Fig 13 shows an increase in throughput achievement of the TDF-ENC and SDF-ENCODT schemes after employing the AdaBoost ENC to reconfigure UAV sensing time and ODT selection. The TDF, SDF, TDF-ENC, and SDF-ENCODT results further confirm throughput achievements at *r*_2_ than *r*_1_, where the SDF and SDF-ENCODT dominate the other schemes.

Similarly, Fig 14 shows the normalized throughput for *v*_1_ (10m/s) and *v*_2_ (20m/s) with fixed radius *r* (800m), which offers higher throughput for *v*_2_ as compared with throughput at *v*_1_. The SDF-ENCODT throughput can dominate the other schemes followed by the TDF-ENC scheme.

For the SDF scheme (SDF1 and SDF2) in Fig 13 as the SNR increases, the normalized throughput generally improves. However, SDF2 (*r*=300m) consistently outperforms SDF1 (*r*=100m) across all SNR levels, indicating that a larger UAV flight radius leads to better throughput for the SDF scheme. Similar to SDF, TDF2 (*r*=300m) performs better than TDF1 (*r*=100m) at almost all SNR levels, suggesting that a larger flight radius positively impacts TDF’s performance. In Fig 14 at most SNR levels, SDF2 (*v*=20m/s) outperforms SDF1 (*v*=10m/s), indicating that higher UAV flight velocities generally lead to better throughput for the SDF scheme. For TDF, the impact of flight velocity is less consistent than in the SDF scheme. TDF1 and TDF2 show similar performance trends across SNR levels, but the improvement in throughput due to higher speed is not as pronounced as in the SDF scheme. Comparison of SDF and TDF schemes results in Figs 13 and 14 show that the SDF scheme (SDF1 and SDF2) tends to achieve higher normalized throughputs than the TDF scheme (TDF1 and TDF2) across different SNR levels and flight velocities. In both SDF and TDF schemes, the versions with ENC and OTD thresholding SDF-ENCOTD and TDF-ENC tend to outperform their counterparts.

Next, the average number of decisions consumed by the simple and proposed virtual CSS schemes in making the global decision is shown in Figs 15–24.

**Fig 15:**
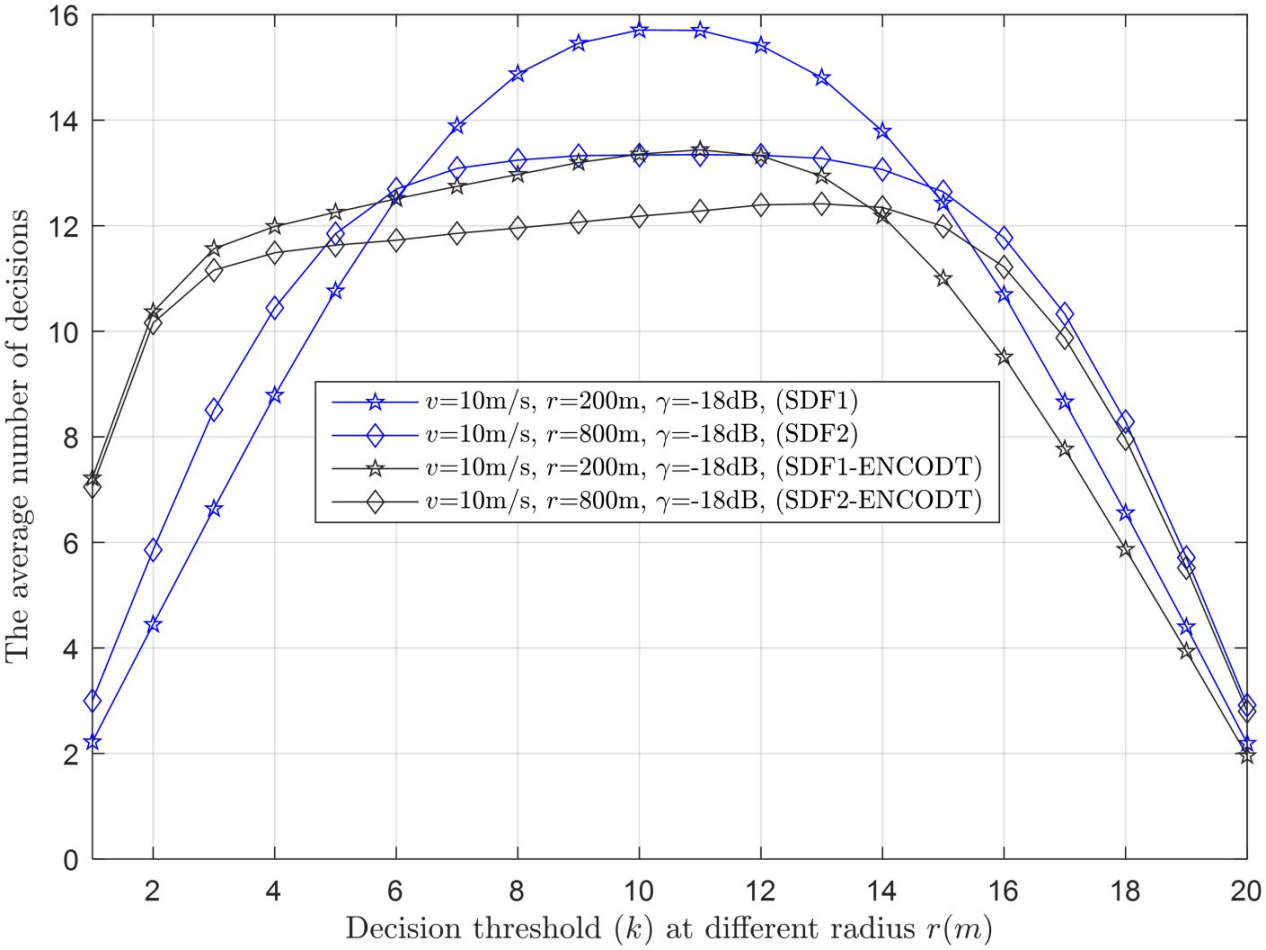
Average decisions vs. decisions threshold at *r* (200m, 800m).

**Fig 16:**
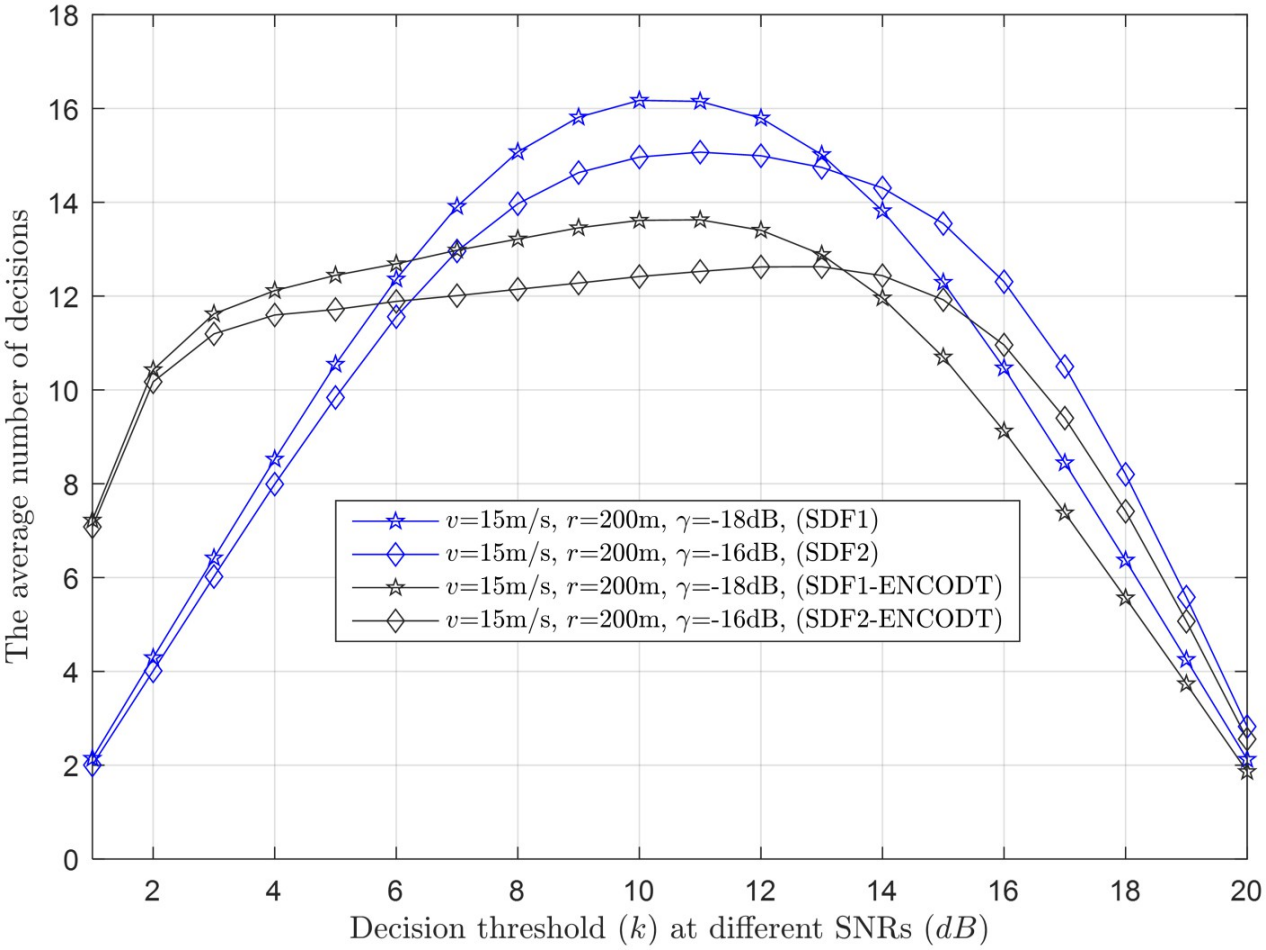
Average decisions vs. decisions threshold (*k*) at *γ* (-18dB, -16dB).

**Fig 17:**
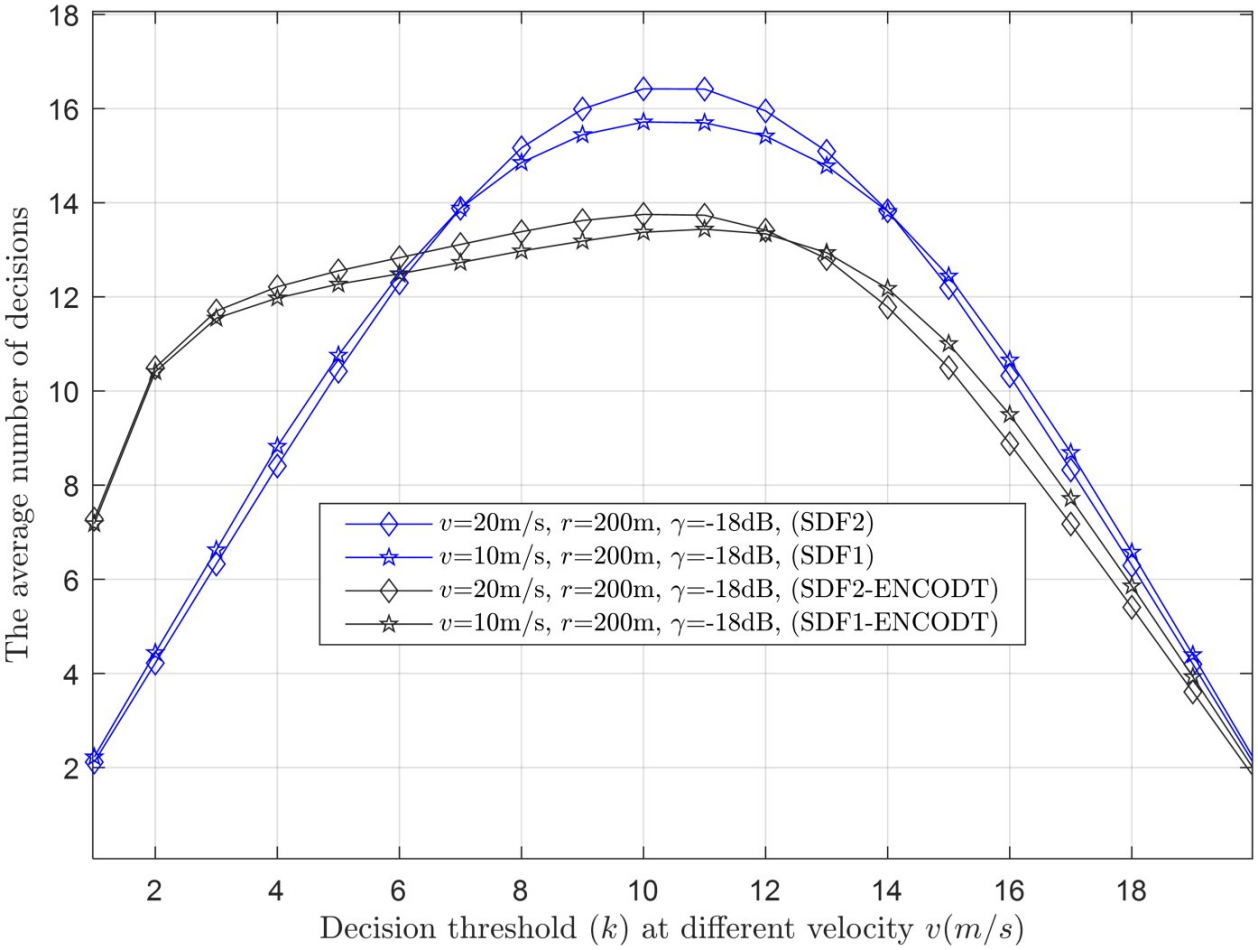
Average decisions vs. decisions threshold at *v* (10m/s, 20m/s).

**Fig 18:**
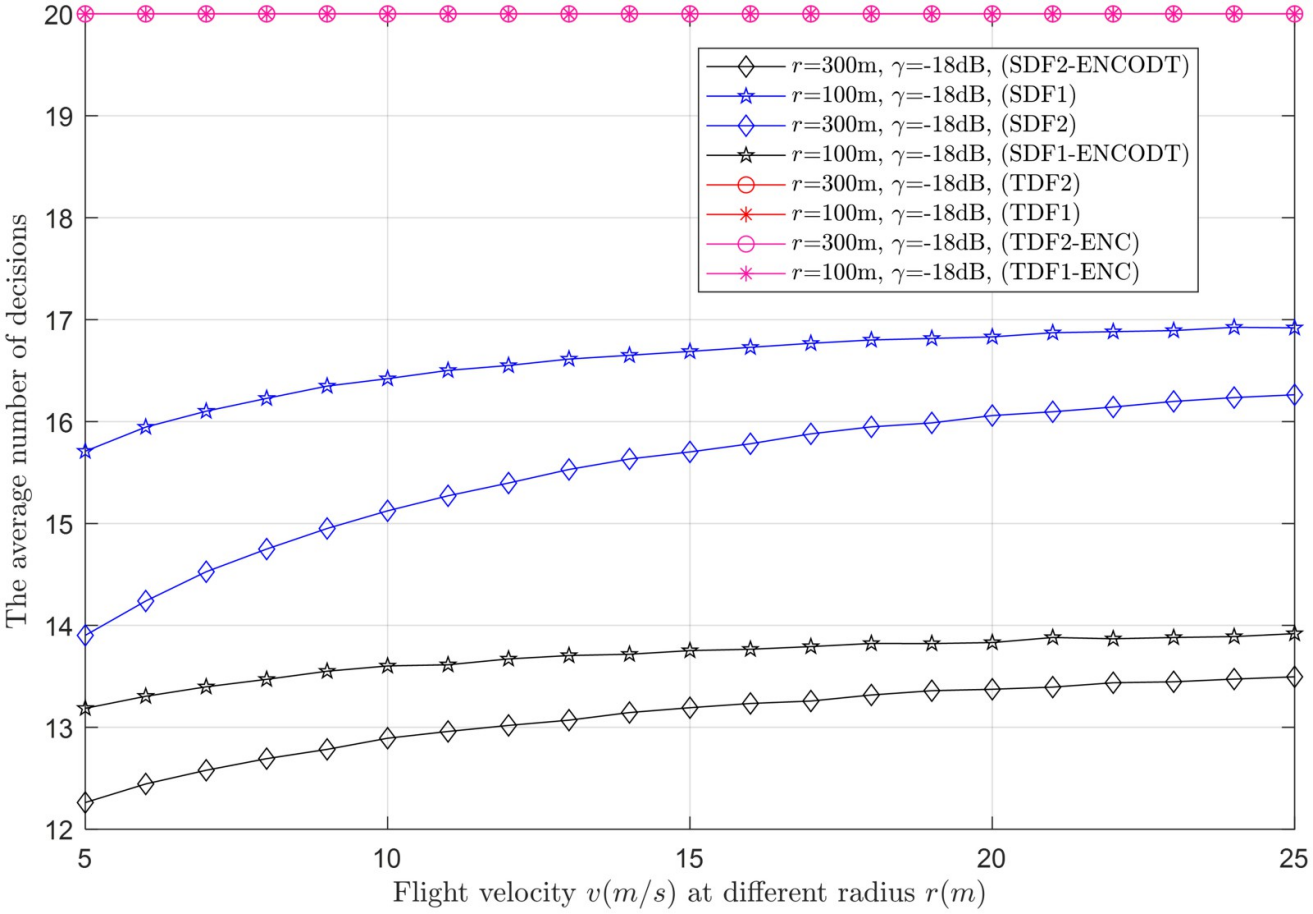
Average decisions vs. flight velocity at *r* (100m, 300m).

**Fig 19:**
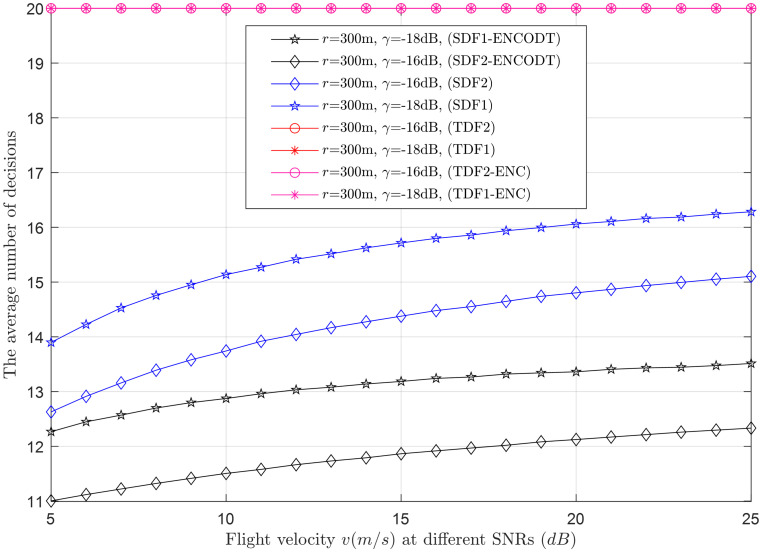
Average decisions vs. flight velocity at *γ* (-18dB, -16dB).

**Fig 20:**
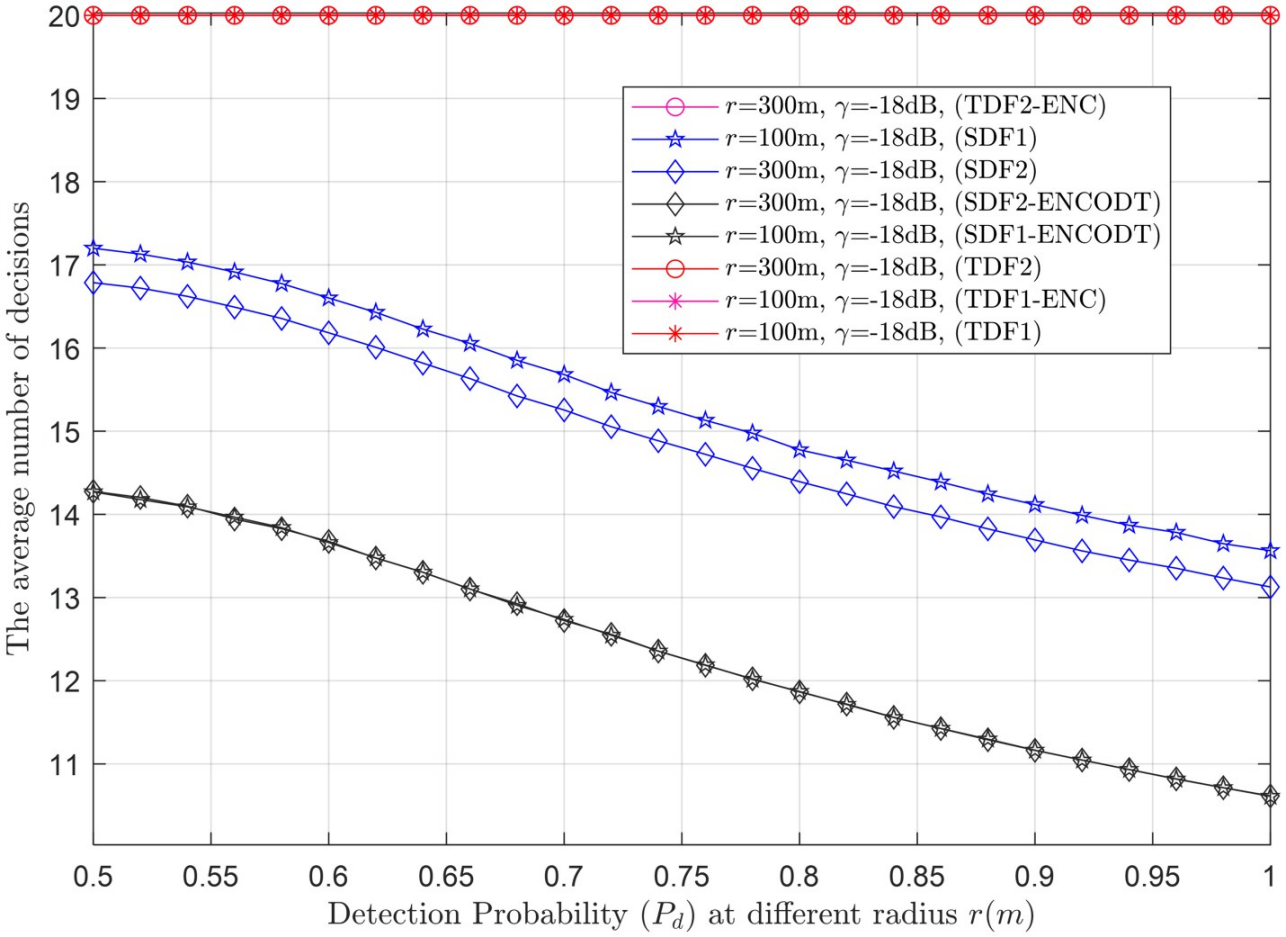
Average decisions vs. detection probability at *r* (100m, 300m).

**Fig 21:**
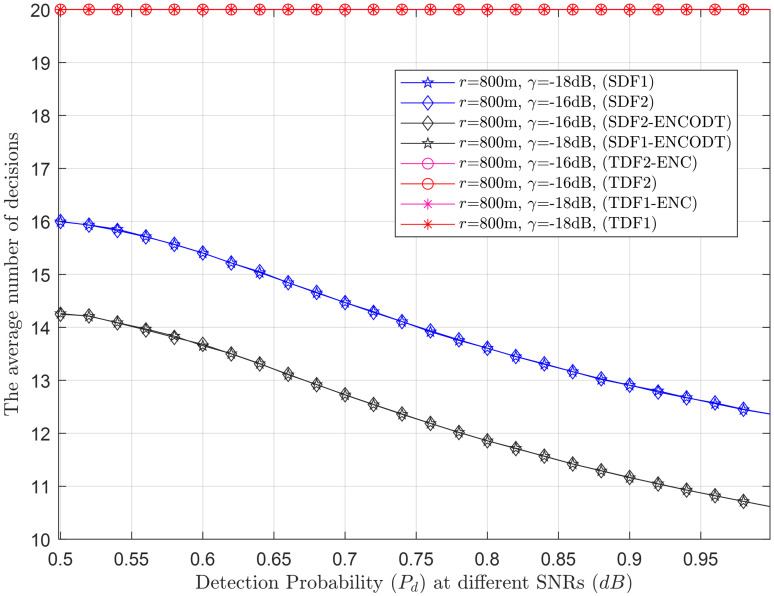
Average decisions vs. detection probability at *γ* (-18dB, -16dB).

**Fig 22:**
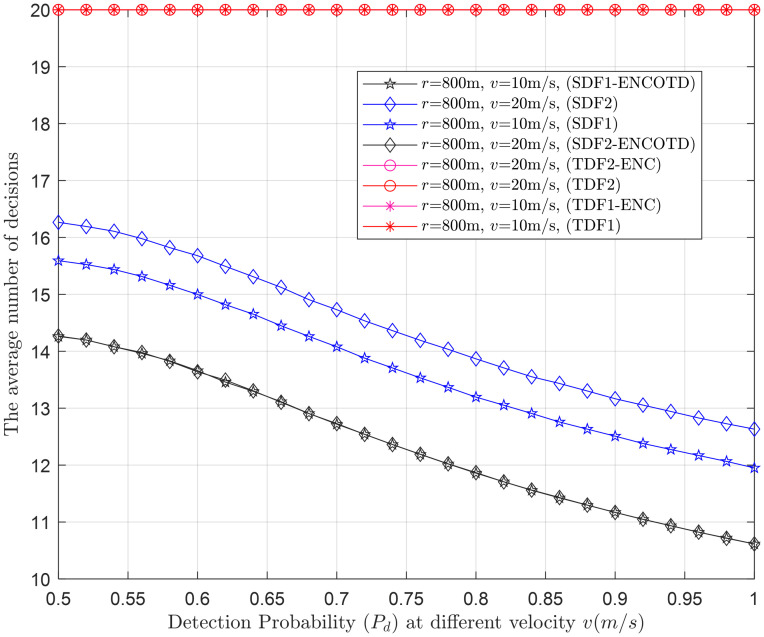
Average decisions vs. detection probability at *v* (10m/s, 20m/s).

**Fig 23:**
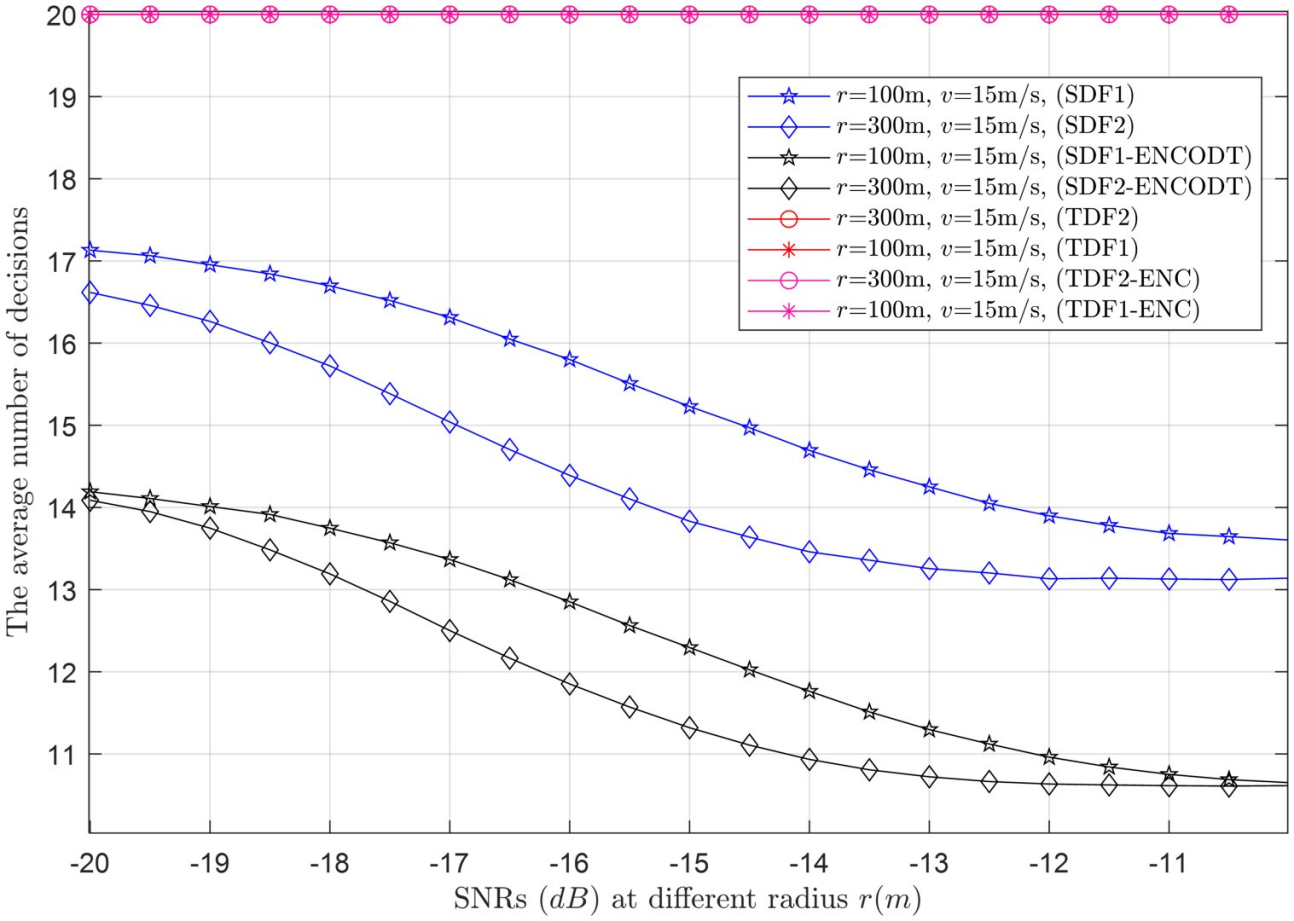
Average decisions vs. SNRs at *r* (100m, 300m).

**Fig 24:**
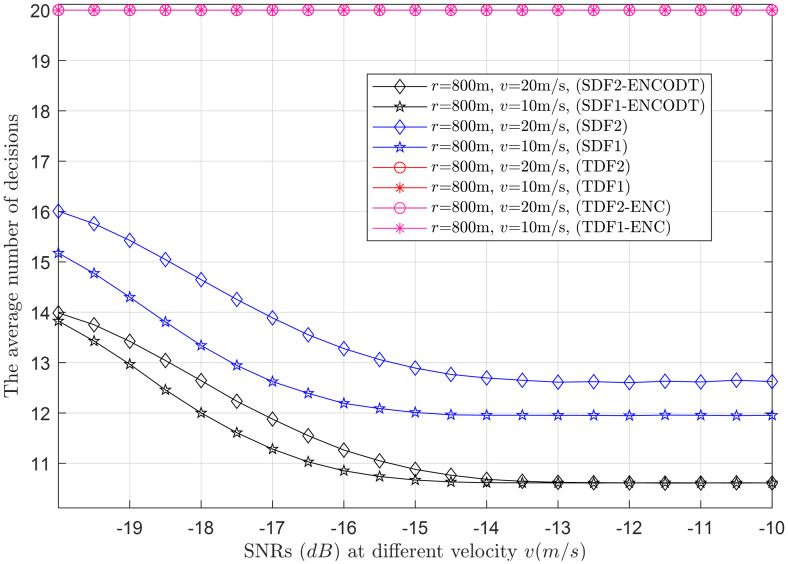
Average decisions vs. SNRs at *v* (10m/s, 20m/s).

### 3.3 Average number of decisions

This section shows a performance comparison of the proposed SDF and SDF-ENCODT schemes in the form of an average number of decisions at different levels of UAV characteristics, such as flight velocity, detection probability, and SNRs in Figs 15–24. The TDF rule is observed to have a fixed number of average decisions because of its constant average decision threshold against varying UAV parameters. Therefore, the section has briefly discussed and compared simple SDF and SDF-ENCODT schemes.

This section provides clear evidence that as *k* increases, the average number of decisions increases and decreases, as demonstrated in Figs 15–17. Higher velocity or a larger sensing radius leads to faster throughput growth in the early stage, followed by a slower growth rate in the later stage. Therefore, the optimal decision rule is *k* = *N* for the simple SDF and SDF-ENCODT schemes that achieve the maximal throughput and best performance. Similarly, increasing flight velocity and SNRs has shown different impacts on the average number of decisions in the SDF-ENC and SDF-ENCODT schemes. The average decisions of the sequential scheme increase with increasing velocity and decrease with increasing SNRs and rising demands of the detection probabilities.

#### 3.3.1 Average decision relation with a decision threshold

Fig 15 shows the average number of decisions against variation in decision threshold (*k*) at different levels of the flight radius *r*_1_ (200m) and *r*_2_ (800m). This indicates that the SDF-ENCODT scheme consumes fewer decisions than the simple SDF scheme at *r*_1_ and *r*_2_. The SDF-ENCODT consumes more average decisions until the decision threshold (*k*=5). The proposed scheme can beat the other method as it passes the threshold. The SDF-ENCODT also shows higher average decisions at *r*_1_ than at *r*_2_ until the decision threshold (*k*=14) and is reversed as it passes *k*=14.

Fig 16 shows a similar performance of the proposed scheme with fewer average decisions at *γ*_1_ (-18dB) and *γ*_2_ (-16dB) as the decision threshold passes *k*=5. The SDF-ENCODT consumes higher average decisions for *γ*_1_ until it reaches the decision threshold *k*=14 in Fig 16. The average decision results in Fig 17 at different flight velocities *v*_1_ (10m/s) and *v*_2_ (20m/s) show minimum decisions for the proposed SDF1-ENCODT and SDF2-ENCODT schemes as compared with SDF1 and SDF2. There is a noticeable increase in the average decisions of the SDF scheme compared to the SDF-ENCODT scheme. The SDF and SDF-ENCODT average decisions converge to an almost similar point as the decision threshold *k* is increased from *k* = 1 reaches *k* = *N*.

In all cases, increasing the decision threshold (*k*) leads to higher average decisions. This behavior is expected as higher *k* values require more evidence before making a decision, resulting in fewer false alarms but potentially more misses. The SDF-ENCOTD scheme offers minimum average decisions compared with a simple SDF scheme as the decision threshold increases. Different combinations of UAV flight radii, SNRs, and flight velocities affect the average decisions differently. However, the overall trend of increasing decisions with higher *k* values remains consistent across most scenarios.

#### 3.3.2 Average decision relation with flight velocity

This section shows the average number of decisions the UAV-SU consumes against the increasing flight velocity at *r*_1_ (100m) and *r*_2_ (300m). The *γ* is fixed as -18dB in Fig 18. The figure shows that as the velocity increases, no change is seen in the average decisions of TDF and TDF-ENC because of their constant average decisions. Consistent and high growth is visible in the average number of decisions of the simple SDF compared with the SDF-ENCODT scheme. The simple TDF and TDF-ENC schemes show no relation to an average number of decisions for the increasing flight velocities and consume consistently high average decisions. The proposed SDF-ENCODT has the minimum number of average decisions out of these schemes at *r*_1_ (100m) and further reduces at *r*_2_ (300m), producing high normalized throughput because of minimum average decisions expanded for sensing.

Fig 19 collects the average sensing decisions against varying flight velocities at *γ*_1_ and *γ*_2_ and fixed radius *r* (300m). This figure shows that the average number of decisions reduces as SNRs increase from *γ*_1_ to *γ*_2_. This result is due to the combination schemes’ quick response to decide the channel availability in case of high SNRs. The result in Figs 18 and 19 confirms that for a given *γ* and radius *r*, increasing flight velocity enables UAVs to observe more sensing slots, leading to more average decisions.

#### 3.3.3 Average decision relation with detection probability

Figs 20–22 illustrate the average decision relation with varying target detection probabilities. The results show that as the target detection probability increases, the average number of decisions of the SDF and SDF-ENCODT schemes reduces except for the TDF, where they always encounter a fixed number of judgments because of no relation to the number of decisions. These figures show that TDF schemes have consistently high total average decisions that do not change with the target detection probability in the network. Fig 20 shows that the simple SDF average decisions get lower as the required detection probability increases for *r*_1_ (100m) and *r*_2_ (300m). Lower average decisions could be seen for the SDF at *r*_2_ (300m) and then at *r*_1_ (100m). The SDF-ENCODT offers minimum average decisions than SDF and TDF schemes showing fewer impacts of the changing flight radius *r*.

Fig 21 shows average decisions for increasing detection probability at *γ*_1_=-18dB and *γ*_2_=-16dB. The figure shows that the average decisions of the SDF are similar at *γ*_1_ and *γ*_2_ and remain identical at both SNRs. The average decisions of the SDF are also similar for both SNRs in Fig 21 and get down compared with Fig 20.

In Fig 22, the radius is kept at 800m, and average decisions are measured for the UAV flight velocities *v*_1_ and *v*_2_. The figure shows that the TDF and TDF-ENC average decisions are always constant at all levels of the target detection probabilities. This indicates that the average decisions are higher for the case of *v*_2_ than *v*_1_ for the simple SDF scheme and decrease as the target detection probability increases. The results in Figs 20–22 have shown similar average decisions of the SDF-ENCODT scheme at different levels of the flight radius, SNRs, and flight velocities.

The SDF schemes tend to become more conservative in making decisions as the demanded detection probability increases. This suggests that as you require a higher probability of correctly detecting the target, the schemes are less likely to declare a target presence unless they are very confident. The TDF schemes always consider fixed average decisions (20) regardless of the demanded detection probability. The SDF-ENCOTD schemes lower the simple SDF decisions, indicating that ensembling and thresholding significantly alter the decision behavior in this specific scenario.

#### 3.3.4 Average decision relation with SNRs

This section collects the average decisions against the *γ* variations at two different levels of the UAV flight radii (*r*_1_, *r*_2_) and velocities (*v*_1_, *v*_2_). As expected, the TDF and TDF-ENC produce consistently high average decisions, while the average decision of both SDF and SDF-ENCODT reduces in Figs 23 and 24. Fig 23 shows that increasing the flight radius from *r*_1_ to *r*_2_ for the given target SNRs reduces the average decisions of both SDF and SDF-ENCODT schemes. The average decisions of the SDF-ENCODT scheme are much lower than the SDF scheme at both flight radii.

The average decisions of the SDF and SDF-ENCODT schemes further get down for the case of *v*_1_ and *v*_2_ in Fig 24 when the flight radius *r* (800m) is constant. Comparing these results, it is also evident that the average decisions of the SDF and SDF-ENCODT reduce to a much lower limit than in Fig 23. The results also indicate that an increased flight radius reduces the average decisions for a given SNR. In contrast, an increase in flight velocity decreases the average decisions for sequential decisions.

Fig 23 shows the average decisions for SDF1 range from approximately 17.13 to 13.60, while SDF2 average decisions range from around 16.62 to 13.14 as the SNR increases from -20dB to -10dB. This indicates that as the SNR increases, the average decision decreases, making it easier for the system to make accurate decisions. TDF and TDF-ENC average decisions are always 20, regardless of the SNR. This suggests that TDF and TDF-ENC have a fixed decision-making capability, regardless of signal strength. Similarly, SDF1-ENCOTD average decisions range from approximately 14.19 to 10.65 and SDF2-ENCOTD range from 14.09 to 10.61 as the SNR increases. In Fig 24 the average decisions for SDF1 range from approximately 15.17 to 11.95 as the SNR increases. Similar to the previous scenario, it shows increased performance with higher SNR levels. The average decisions for SDF2 are similar to SDF1, ranging from around 16.01 to 12.62 as the SNR increases. The average decisions for SDF1-ENCOTD range from approximately 13.83 to 10.61 as the SNR increase. It performs similarly to the previous scenario’s SDF1-ENCOTD, with increased performance at higher SNR levels. Similarly, the average decisions for SDF2-ENCOTD range from around 13.99 to 10.61 as the SNR increases. The SNR plays a crucial role in the performance of the detection schemes. As SNR increases, the average decisions for all methods reduce, indicating minimum decisions while making cooperative decisions. The UAV flight radius and velocity do not significantly impact the decisions, as the average decisions remain constant across different scenarios for TDF and TDF-ENC schemes. The TDF and TDF-ENC schemes consistently show constant average decisions with average decisions of 20, regardless of the SNR and other parameters. This suggests that these schemes have a consistent behavior to noise and variations in UAV flight characteristics. The SDF and SDF-ENCOTD schemes show a gradual decrease in the average decisions as the SNR increases. This suggests that they are more adaptive to noise and challenging environmental conditions.

### 3.4 Cooperative sensing decision

**Fig 25:**
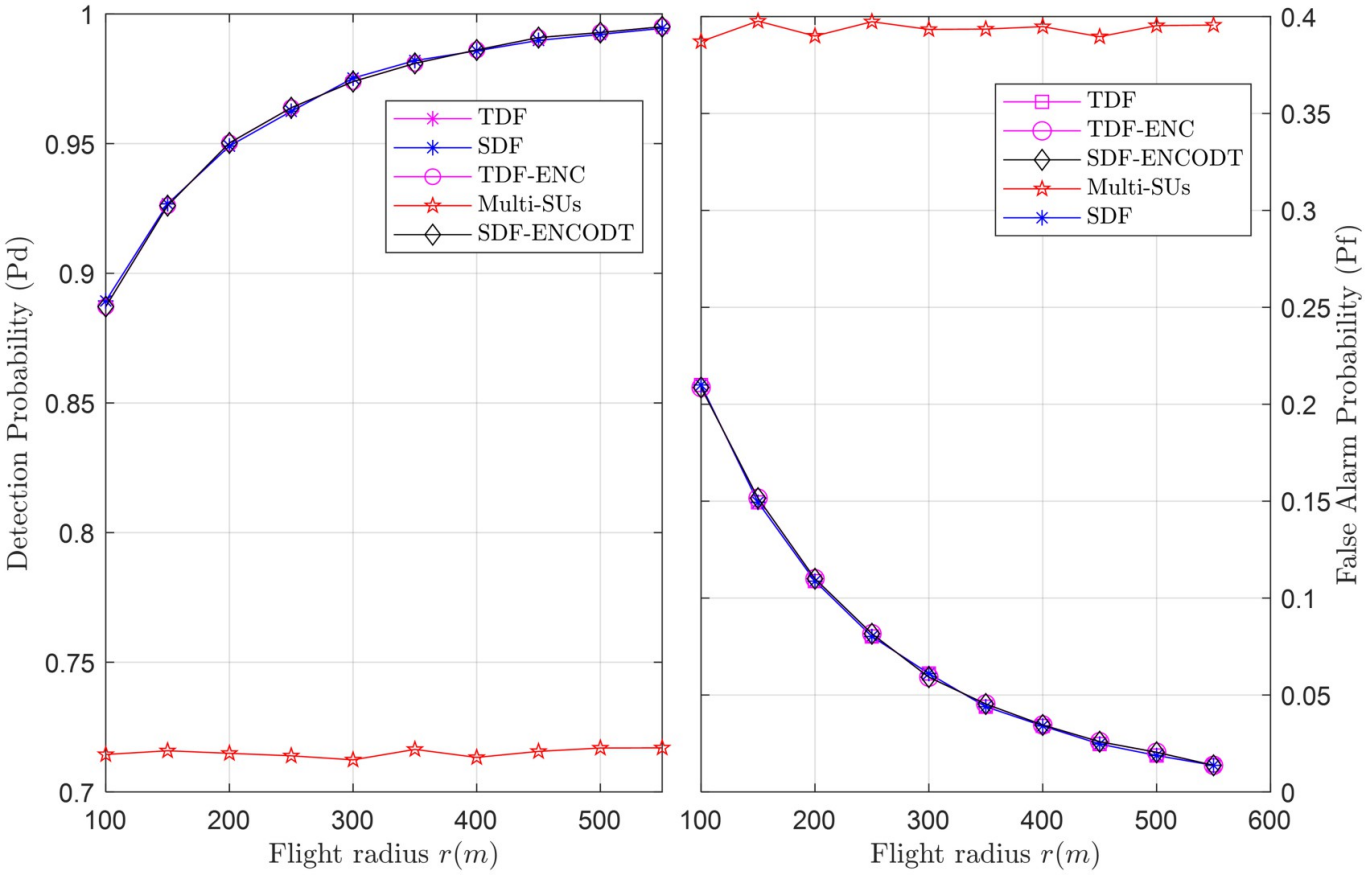
Cooperative decision vs. flight radius (*r*).

**Fig 26:**
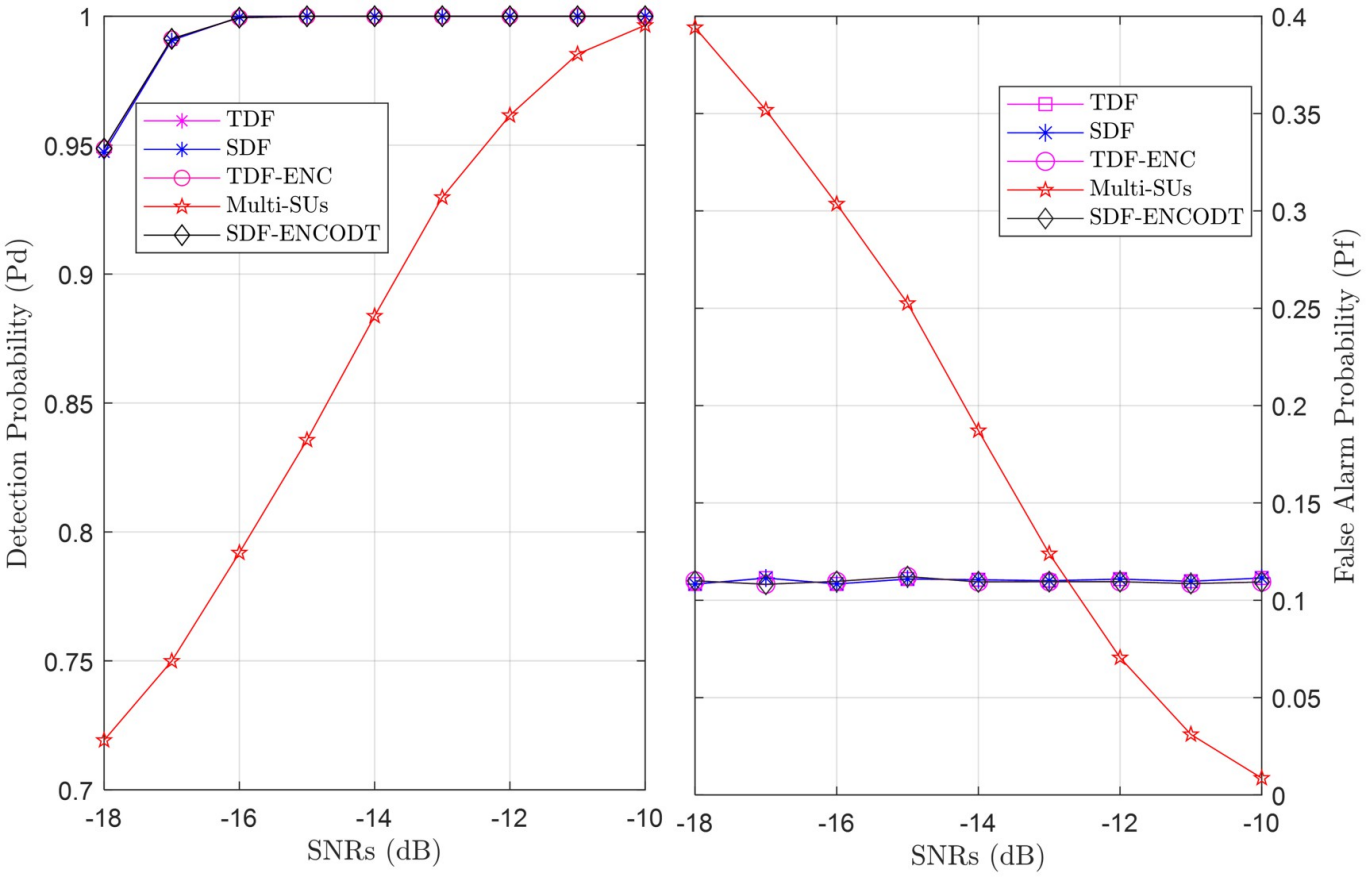
Cooperative decision vs. SNRs (dB).

**Fig 27:**
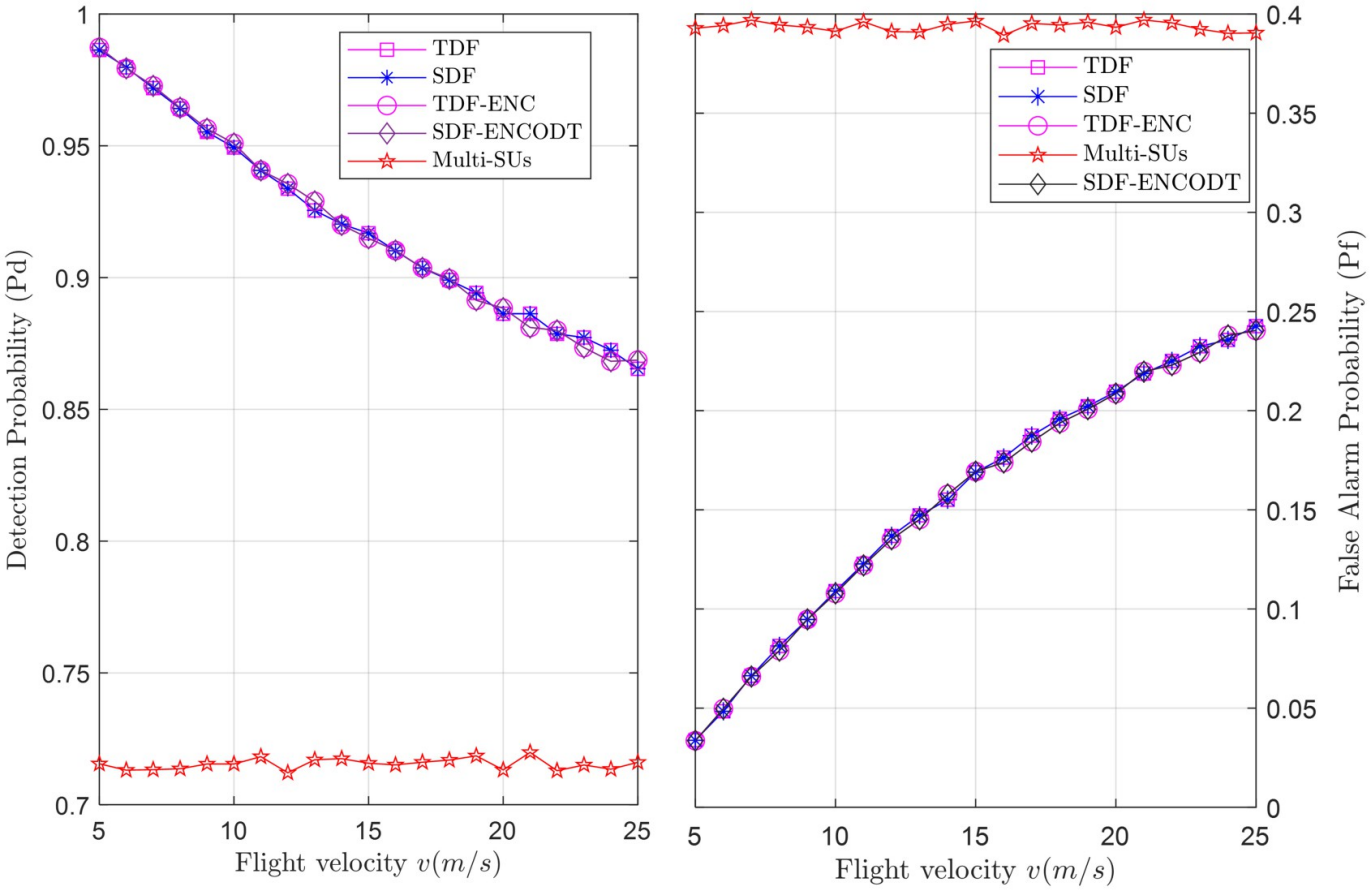
Cooperative decision vs. flight velocity (*v*).

To compare sensing reliability, Figs 25–27 show the global detection and false alarm probability results of the SDF, TDF, TDF-ENC, and SDF-ENCOTD schemes at different levels of flight radius, flight velocity and SNRs. The results are compared with the multi-SUs environment, including 16 cooperative SUs randomly distributed at different geographical positions that report the PU channel sensing statistics to the FC for final decision. Figs 25 and 26 show an increase in detection probability and a decrease in false alarm probability for the UAV-based virtual CSS schemes compared with multiple SUs environments as the flight radius of the UAV and received SNRs increases. Similarly, Fig 27 illustrates a decrease in detection probability and an increase in false alarm probability as the flight velocity increases. As expected, these global sensing results produce similar detection and false alarm results for the TDF and SDF-based virtual cooperative schemes.

## 4 Conclusions

Integrating UAVs with CR technology reduces the spectrum scarcity issues for UAV communication. However, the multi-user CSS is not an optimal choice in the UAV-CRN due to UAVs with limited power availabilities. This work proposes a virtual CSS scheme for UAVs in CR networks, which employs a single flying UAV to perform CSS. The proposed scheme utilizes SDF to add mini-slot local decisions and make a final decision using the *k* − *in* − *N* majority voting rule. The TDF scheme with a fixed decision threshold is not suitable for the UAV network due to its high average decisions. At first, the TDF and SDF mini-slots sensing radians are configured using ENC with the AdaBoost ensembling method. Then the optimal decision threshold is selected for the proposed SDF to compare the sum of the sequential decisions with an adjustable threshold using the *k* − *in* − *N* rule. Normalized throughput and average decision results are obtained at different levels of the flight velocities, decision threshold, SNRs, and target detection probabilities. As a result, the TDF-ENC and SDF-ENCODT have improved throughput and energy efficiency compared with simple TDF and SDF schemes.
